# Access to quality trauma care after injury in Pakistan: a systematic review and narrative synthesis

**DOI:** 10.1136/bmjopen-2025-101071

**Published:** 2025-12-07

**Authors:** Huba Atiq, Komal Abdul Rahim, Sijal Akhtar Shiekh, Badar Afzal, Zabin Wajidali, Zaheer Babar Chand, Asad Latif, Agnieszka Ignatowicz, Leila Ghalichi, Kathryn Chu, Junaid A Razzak, Justine Davies

**Affiliations:** 1 Centre of Excellence for Trauma and Emergencies & Department of Anesthesiology, The Aga Khan University, Karachi, Sindh, Pakistan; 2 Applied Health Sciences, College of Medicine and Health, University of Birmingham - Edgbaston Campus, Birmingham, England, UK; 3 Centre of Excellence for Trauma and Emergencies, The Aga Khan University, Karachi, Sindh, Pakistan; 4 Department of Emergency Medicine, The Aga Khan University, Karachi, Sindh, Pakistan; 5 Anaesthesiology, The Aga Khan University Faculty of Health Sciences, Karachi, Sindh, Pakistan; 6 University of Birmingham, Birmingham, West Midlands, UK; 7 University of Birmingham; 8 Applied Health Research, University of Birmingham, Birmingham, UK; 9 Global Health, Stellenbosch University Faculty of Medicine and Health Sciences, Cape Town, South Africa; 10 Department of Emergency Medicine, Weill Cornell Medicine, New York, New York, USA; 11 Global Health, University of Birmingham, Birmingham, UK

**Keywords:** Trauma, Quality in health care, TRAUMA MANAGEMENT, ACCIDENT & EMERGENCY MEDICINE, Health Services Accessibility

## Abstract

**Abstract:**

**Objectives:**

To conduct a systematic review and narrative synthesis to identify barriers, facilitators and pre-existing interventions and describe the current status of initiatives/interventions aimed at improving access to quality trauma healthcare after injury in Pakistan.

**Design:**

Systematic review and narrative synthesis

**Data sources:**

MEDLINE (Ovid), Embase (Ovid), Web of Science (Clarivate Analytics), Cochrane (Wiley), Scopus and ProQuest, as well as grey literature.

**Eligibility criteria:**

Full-text peer-reviewed publications, including cross-sectional studies, cohort studies, case-control studies, randomised controlled trials and qualitative studies published in English from January 2013 to December 2023.

**Data extraction and synthesis:**

Two independent reviewers used a standardised tool to extract data variables to Excel. The quality of the included studies was evaluated using the CASP checklist. The barriers, facilitators and pre-existing interventions were mapped using the four delays framework, the Institute of Medicine (IOM) quality domains and the WHO health systems building blocks. The data were synthesised narratively to improve access to quality trauma care in Pakistan. This review was reported following the Preferred Reporting Items for Systematic Reviews and Meta-Analyses (PRISMA) reporting guidelines.

**Results:**

The review included 20 studies. 19 studies reported 58 barriers to access to quality care. Six studies reported 20 facilitators, and eight studies described initiatives or interventions aimed at improving access to quality trauma healthcare after injury. According to the four delays framework, the receiving care stage of access to care was primarily studied in 16 studies, which identified 37 barriers and 13 facilitators across 5 studies. Regarding the quality of care according to IOM domains, the effectiveness of quality trauma care after an injury was studied in 15 studies, which identified 19 barriers and 10 facilitators across four studies. According to the WHO health system building blocks, most studies (n=15) described challenges in healthcare service delivery, with these 15 studies identifying 23 barriers and 3 studies identifying 4 facilitators.

**Conclusion:**

Our findings highlighted the scarcity of available literature, identified barriers and facilitators and pre-existing interventions, which informed the need to develop feasible, sustainable and contextually relevant interventions to improve access to quality trauma care after injury in Pakistan.

**PROSPERO registration number:**

CRD42024545786

**STRENGTHS AND LIMITATIONS OF THIS STUDY**

- This systematic review synthesises evidence on access to quality trauma care in Pakistan using predefined frameworks to guide data extraction and analysis.
- A comprehensive search strategy was applied across multiple databases and grey literature sources following systematic review standards.
- Standardised Critical Appraisal Skills Programme checklists were used to appraise study quality, ensuring transparent assessment of methodological strengths and weaknesses.
- The review was limited to studies published in English within the last 10 years, which may have excluded some relevant evidence.
- Most included studies were cross-sectional with limited methodological rigour, and the scarcity of interventional studies may affect the robustness of the conclusion.

## Background

 Injury significantly contributes to death and disability worldwide, ranking sixth and seventh among the causes of these outcomes, respectively.^1^ Globally, 50 million individuals are left disabled every year following an injury.^2^ The burden of injury is disproportionately higher in low- and middle-income countries (LMICs) than in high-income countries, and it is estimated that 2 million deaths per year could be averted through the provision of adequate trauma care in LMICs.^2 3^

With a high burden of injuries affecting the population, trauma care in Pakistan is a crucial problem. According to the Global Burden of Disease (GBD), injuries account for 6.56% of total deaths and 7.18% of total disability-adjusted life years (DALYs) in Pakistan.^4^ Several national and subnational surveys have been done in Pakistan to investigate the prevalence and outcomes of injuries. The Pakistan Demographic and Health Survey (PDHS) 2020 reported that road traffic accidents, falls, drowning, homicide, suicide and burns cause 4% of deaths in Pakistan, and death rates due to road traffic injuries are 12 per 1000.^5^ Among this growing epidemic of injuries, road traffic accidents are ranked ninth in the leading causes of death and disability nationally as of 2021.^6 7^ It is important to note that most of the injury-related deaths are preventable and occur in the economically productive age group 15–64 in Pakistan, with 18.3% of deaths and 13.5% of disability-associated life years attributed to injuries.^1 4 8^

Injury prevention is undoubtedly needed. However, providing access to quality trauma care to those who are inevitably injured in Pakistan is likely to remain a challenge, especially in a nation with a demographically significant young population living below the poverty line and facing political instability, terrorism and frequent natural disasters such as earthquakes and floods.^8^ Additionally, growing urbanisation, increasing utilisation of unsafe vehicles or motors, travel in commercial or public buses and incompliant behaviour towards following traffic rules contribute to road traffic injuries in the country.^6 9^ Improved healthcare infrastructure and trauma centres are needed in Pakistan to ensure prompt access to quality trauma care services.^1^

There are challenges in developing evidence-based interventions and policies around access to quality trauma care in the country. Injuries still remain a part of the country’s non-communicable diseases (NCD) policies rather than being recognised as a distinct disease burden.^10 11^ This limits the development of specific divisions and policy implementors tailored to the unique context of injuries, separate from NCDs. The scarcity of adequately trained healthcare providers, such as trauma surgeons, emergency physicians, anaesthesiologists and nurses, adds to Pakistan’s trauma treatment hurdles.^10^ These challenges reflect the fragile and non-integrated healthcare system in the country and are consistent with the findings of the Lancet Global Health Commission on High-Quality Health Systems. The Commission estimated that each year, 8.6 million people in LMICs die from conditions that should be treatable within health systems. Approximately 3.6 million of these deaths are due to lack of access, while 5.0 million (nearly 60%) occur among those who accessed care but received poor-quality services.^12^

Achieving better patient healthcare outcomes through access to quality trauma care is a multifaceted and complex phenomenon that requires the integration of an injury care continuum throughout a patient’s journey, from scene to prehospital care to in-hospital care and rehabilitation, as captured by the four-delay framework: seeking care, reaching care, receiving care and remaining in care.^13^ Providing ‘the right care at the right time’ to patients should also be done while ‘responding to the service users’ needs and preferences while minimising harm and resource waste’.^14^ These aspects are captured by the Institute of Medicine’s (IoM) six quality domains: effectiveness, safety, patient-centredness, timeliness, equity and efficiency.^15^ In turn, the delivery of IOM-quality health outcomes depends on providing five critical foundational elements of healthcare systems, as captured by the WHO building blocks, to ensure high-quality workforce excellence across healthcare services, safe and effective use of essential resources, information technology and constant financial and leadership governance support.^14 16^

Hence, we mapped the literature on access to quality trauma care in Pakistan to facilitate a clear understanding of barriers, facilitators and challenges by using the ‘four delays’ framework, the ‘IOM quality domains’ and the ‘WHO health systems building blocks’. This systematic review and narrative synthesis uses these three frameworks to provide insights into Pakistan’s existing trauma healthcare system, including any initiatives or interventions implemented to improve access to quality trauma care in the country.

### Objective

To conduct a systematic review and narrative synthesis to identify barriers, facilitators and pre-existing interventions and describe the current status of initiatives/interventions aimed at improving access to quality trauma healthcare after injury in Pakistan.

## Methods

We followed the Preferred Reporting Items for Systematic Reviews and Meta-Analyses (PRISMA) 2020 guidelines.^17^ This protocol has been registered with PROSPERO (CRD42024545786).

### Eligibility Criteria

We included all full-text peer-reviewed publications, including cross-sectional studies, cohort studies, case-control studies, randomised controlled trials and qualitative studies. We used a detailed search strategy to find studies published in English from January 2013 to December 2023. The study’s inclusion and exclusion criteria are outlined in table 1.

**Table 1:** Inclusion and Exclusion Criteria

	Inclusion criteria	Exclusion criteria
Population	All patients who have physical trauma (injury) after road traffic accidents (RTA), falls, drowning, burns, bites, poisoning, violence/physical abuse, firearm/gunshot injuries, wounds and polytrauma	Non-trauma patients Mental trauma patients
Intervention/ Exposure	Access to healthcare after trauma, including getting care in the community, pre-hospital, hospital or rehabilitation.	
Comparison	No comparator	
Outcome	The provision of quality trauma care as described in any domain of the Four-Delay Model, WHO health system framework, or Institute of Medicine domains. Outcomes of interventions to improve trauma care services.	Studies that do not describe quality trauma care as described in the inclusion criteria
Type of Study	All peer-reviewed articles, including observational, quantitative, and qualitative studies. Grey literature, including from the Higher Education Commission, the Pakistan Medical Association website and the local thesis repository. It also includes non-published reports, national databases, policy documents and international databases (like WHO).	Educational studies Case series/reports/reviews Press articles/Blog entries/social media. Commentaries/ Editorials
Timeframe	2013–2023	Before 2013
Setting	Country: Pakistan Trauma care provided at the following settings: Accident scene in the community During prehospital transport In the hospital After discharge from the hospital to home Rehabilitation units	Other countries

### Search Databases and Search Strategy

We electronically searched the following databases: MEDLINE (Ovid), Embase (Ovid), Web of Science (Clarivate Analytics), Cochrane (Wiley), Scopus and ProQuest. However, given our consideration that many interventions are not written up as formal scientific publications, especially in Pakistan, it is important to conduct a comprehensive review that also considers grey literature. The grey literature was searched using the first 20 pages of Google Scholar and by targeting searches of local websites, such as the Higher Education Commission, the Pakistan Medical Association website and local thesis repositories relevant to providing trauma care in Pakistan. We also reviewed non-published reports, national and international databases (eg, WHO), guidelines and factsheets identified by the authors. We used the PICOST framework to develop our search strategy, as found in online supplemental file 1: annexure A.^18^ The strategy includes keywords and subject matter headings addressing healthcare provision following ‘physical trauma (also called injury),’ ‘access to healthcare after trauma’ and ‘quality of trauma care’. EndNote was used to identify duplicates, and an article reference search was done to review relevant articles.

### Identification of studies

Two authors (HA and KAR) reviewed the eligible articles. Each author independently reviewed a random 20% of the articles to ensure consistency in applying the inclusion and exclusion criteria. Any discrepancies between their assessments were discussed in a consensus meeting attended by three authors (HA, KAR and SAS) to determine the inclusion and exclusion of each article. The goal was to achieve a 90% agreement. Once agreement was reached, the researchers divided the remaining articles for screening titles and abstracts. In cases of disagreement during the review process, a third reviewer (SAS) provided a final assessment. We used the same approach to screen the full-text article reviews and record reasons for excluding articles from full-text screening. We used Covidence to screen the articles by title and abstract, followed by full-text review and data extraction of eligible articles onto a pre-designed Excel sheet.

### Risk of Bias

We aimed to conduct a comprehensive mapping of available evidence and generate insights, rather than performing a meta-analysis of effect sizes. Quality appraisal was performed using standardised criteria from the Critical Appraisal Skills Programme (CASP) Quality Appraisal Checklist. We used a cross-sectional, cohort and qualitative CASP checklist and scored accordingly, according to the CASP critical score as follows: if the criterion was completely met, 2 points; if the criterion was partially met, 1 point and if it was not applicable/unmet/ not mentioned, 0 points. The CASP checklists transparently highlight the methodological strengths and limitations, but all studies were retained to ensure that potentially valuable contextual information was not lost. This approach is consistent with the narrative synthesis methodology, which emphasises inclusivity of diverse sources while accounting for quality in the interpretation of findings (online supplemental file 2: annexure B).

### Data Extraction

We used a standard data extraction tool to collect data onto a Microsoft Excel sheet. Variables included publication information, study methodologies, clinical characteristics, study outcomes using models defined above and, where appropriate, interventions or initiatives introduced to improve access to quality healthcare after injury (table 2). We piloted the data extraction tool on full-text screening. Similar to the methodology used for screening, two independent reviewers (HA, SA) extracted data until 90% of the agreement was achieved, and a third reviewer resolved the disagreement.

**Table 2:** Standardised data collection tool—study variables

Publication information	Study ID, year of publication, first author, last author, citation, author contact information, and journal name.
Study methodologies	Study design, study duration, geographical location (urban/rural), study site (province-wise), study settings (community, pre-hospital, primary care, secondary care, tertiary care, trauma centre, home, rehabilitation), study inclusion and exclusion criteria, study data collection methods, study population and sample size
Clinical characteristics of persons	Age, gender, mechanism of injury and injury type.
Study Outcome	Categories using a four-delay framework (seeking care, reaching care, receiving care, remaining in care), Institute of Medicine (IOM) domains of quality (safety, effectiveness, timeliness, patient-centredness, equity*), and WHO health system building blocks (healthcare workforce, services, financing, leadership and governance, information technology, and access to equipment and medication,
Interventions/Initiatives	Any intervention or initiatives introduced to improve access to quality healthcare after injury and type of intervention/ initiatives.

* We did not include the IOM domain of ‘efficiency’ as avoiding waste/efficient use of resources was not part of our study objective.

### Data Analysis and Synthesis

Quantitative and qualitative data were synthesised in narrative form to address the scope and objectives of this systematic review. Tables, graphs and figures were used to present the results. The systematic review was reported by following the reporting guidelines in the Preferred Reporting Items for Systematic Review and Meta-Analysis (PRISMA) statement.^17^

### Data Availability

This study has not been posted as a preprint.

### Patient and Public Involvement

Patients and the public were not involved in the design, conduct, reporting or dissemination of this systematic review and narrative synthesis.

### Funding

This study is funded by the NIHR Global Health Group on Equitable Access to Quality healthcare for Injured People in Four Low- or Middle-Income Countries: Equi-injury—NIHR Global Health Groups. Grant number: NIHR133135.^19^

## Results

### Included studies

**Figure 1:**
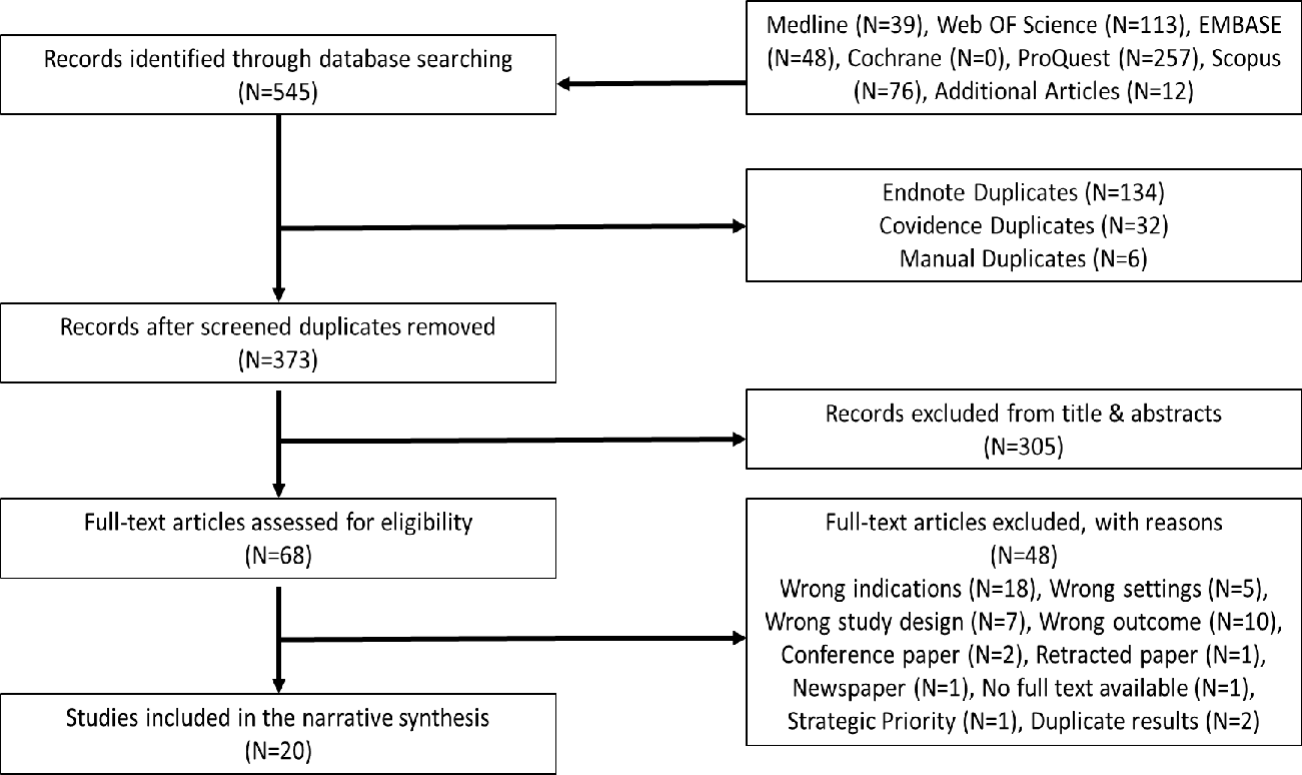
Preferred Reporting Items for Systematic Reviews and Meta-Analyses chart of study screening.

Initial searches identified 545 articles, including 533 from the primary literature search and 12 from the grey literature search. After removing duplicates, 373 articles were eligible for the initial title and abstract screening. Subsequently, the full-text review was conducted for 68 articles and 20 articles were eventually included for data extraction and narrative synthesis (figure 1). 17 of the 20 included studies were from the primary literature search,^120^,^35^ while three were from the grey literature.^36^,^38^

### Study Design Characteristics

The majority of the studies (n=19; 95%) were quantitative,^120^,^33 35^ with 85% (n=17) being observational cross-sectional studies,^120^,^27 29 30 32 33 36^ most of which were conducted prospectively (n=16/19; 89%).^120^,^30 35^ Among these studies (n=19), 58% (n=11) described patient characteristics related to injuries,^2223 26^,^28 30^ while 42% (n=8) focused on facility characteristics,^1 20 21 24 25 29 36 37^ including human resources and infrastructure. The data collection methods employed in these studies include structured questionnaires (n=12; 63%),^20^,^2224^ patient medical records (n=4; 21%),^2731^,^33^ and registries (n=3; 16%)^1 23 28^ (online supplemental file 3: annexure C).

### Study Settings

Of the included studies, 80% (n=16) were from the province of Sindh.^121^,^23 25^ Geographically, urban tertiary care centres (teaching hospitals in Pakistan typically equipped with multidisciplinary specialties and subspecialties) were the setting for 90% (n=18/20) of the studies^120^,^33 35 37 38^ (online supplemental file 3: annexure C).

### Study Population

A total of 11 patient-related studies were included in this systematic review comprising 15 060 participants, with individual study sample sizes ranging from 32 to 6212^2223 27 28 30^,^32 38^ (online supplemental file 4: annexure D). The mean age across nine studies was 34.5 years, with participants ranging from 1 to 89 years of age^2223 26^,^28 30^ (online supplemental file 4: annexure D). Gender distribution was reported in all included studies (n=11), with 11,628 males and 3,294 females.^2223 26^,^28 30^ Information on education level and socioeconomic status was not reported in any of the studies (online supplemental file 4: annexure D). Road traffic crashes (RTCs)^22 23 28 30 32 33 35^ were the most common mechanism of injury, accounting for (n=7; 63%) of the cases reported, followed by gunshot injuries (n=2; 18%),^26 31^ falls (n=1; 9%)^27^ and dog bites (n=1; 9%)^38^ (Annexure C & D).

### Study Outcomes

**Figure 2:**
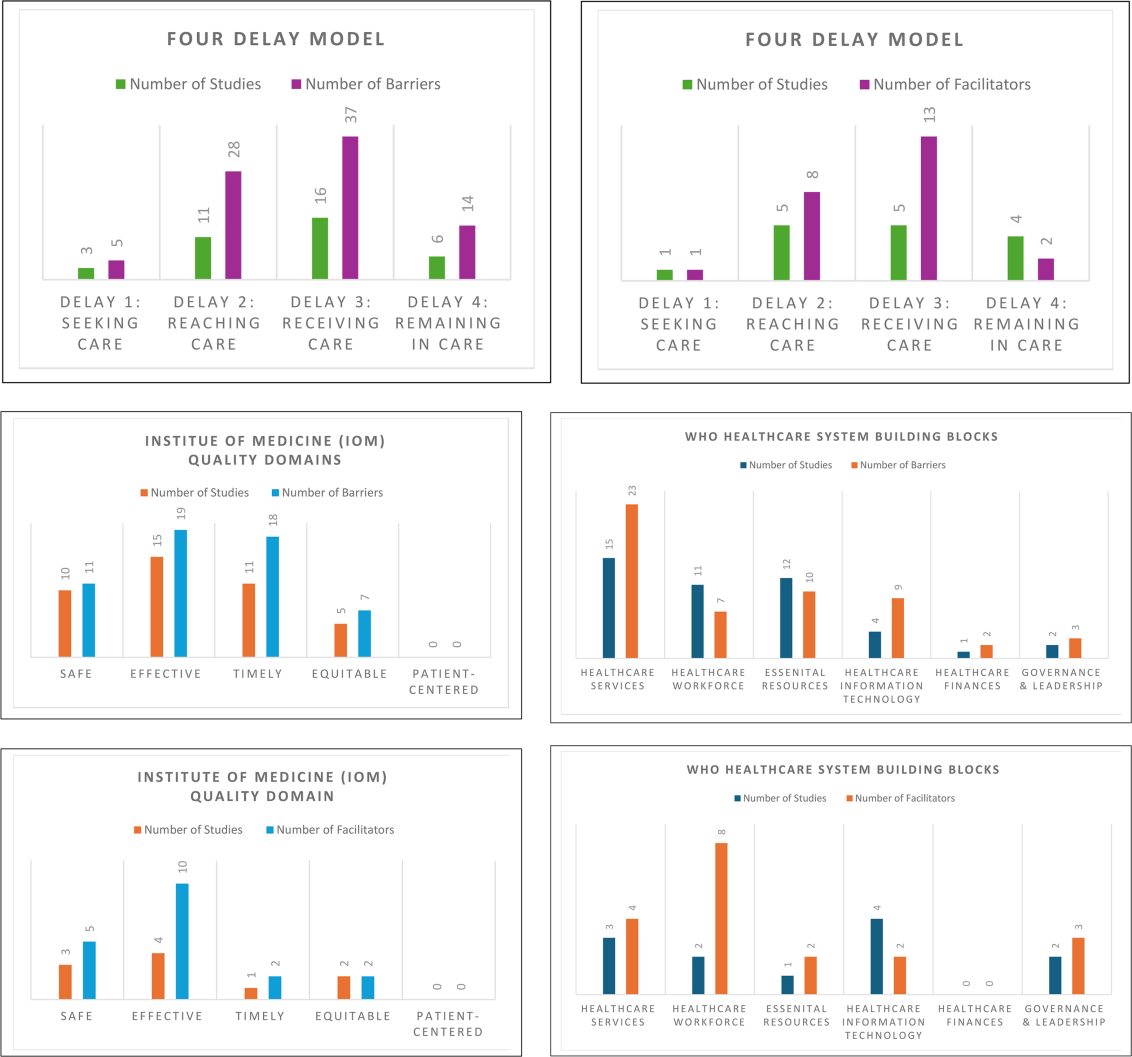
Studies Outcome – Barriers & Facilitators found in studies using the Four-Delay Model, WHO Healthcare System Building Blocks & Institute of Medicine Quality of Care Domain.

19 studies^120^,^30 32^ reported a total of 58 barriers to access to quality care, 6 studies reported a cumulative of 20 facilitators^23 27 28 31 34 38^ and 8 studies described either initiatives or interventions^123 26^,^28 31 34 38^ to improve access to quality trauma healthcare after injury (table 3, figure 2, onlinesupplemental files 3; 58: annexures E, F, G and H).

**Table 3:** Studies Outcome – Barriers & Facilitators found in studies using the Four-Delay Model, WHO Healthcare System Building Blocks & Institute of Medicine Quality of Care Domain

	Barriers			Facilitator		
	Number of Studies (n=19)	Number of Barriers (n=58)	References	Number of Studies (n=6)	Number of Facilitators (n=20)	References
Four-Delay Model						
Delay 1: Seeking care	3	5	(^30 37 38^)	1	1	(^38^)
Delay 2: Reaching Care	11	28	(122 24 26,28 30 32 34 36 38)	5	8	(^23 27 28 34 38^)
Delay 3: Receiving care	16	37	(120,28 30 32 33 35 37 38)	5	13	(^23 27 28 31 38^)
Delay 4: Remaining in care	6	14	(2227,29 35 38)	4	2	(^23 27 28 38^)
Institute of Medicine (IOM) Quality Domains						
Safe	10	11	(2024 26 28 32,34 36)	3	5	(^23 31 34^)
Effective	15	19	(120 22,30 32 36)	4	10	(^23 27 28 34^)
Timely	11	18	(124 27 29 30 32 33 35,38)	1	2	(^23^)
Equitable	5	7	(^21 22 24 35 38^)	2	2	(^34 38^)
Patient-centred	0	0	0	0	0	0
WHO Healthcare System Building Blocks						
Healthcare services	15	23	(121 24,30 32 33 35)	3	4	(^23 31 34^)
Healthcare workforce	11	7	(2022,24 26 28 30 32 33 36 37)	2	8	(^23 34^)
Essential resources	12	10	(2022 24 26 28,30 34)	1	2	(^31^)
Healthcare information technology	4	9	(^27 28 37 38^)	4	2	(^23 27 28 38^)
Healthcare finances	1	2	(^22^)	0	0	0
Governance and leadership	2	3	(^27 34^)	2	3	(^23 34^)

According to the Four-Delay Model, the ‘Receiving Care’ stage of access to care was most often studied, with the identification of 37 barriers spread over 16 studies^120^,^28 30 32 33 35 37 38^ and a total of 13 facilitators reported in five studies combined.^23 27 28 31 38^ ‘Reaching care’ was the subsequent most studied, with 11 studies describing 28 barriers^122 24 26^,^28 30 32 34 36 38^ and 5 studies identifying 8 facilitators.^23 27 28 34 38^ At the ‘maining in care’ stage, 6 studies identified 14 barriers^2227^,^29 35 38^ and 4 studies found 2 facilitators.^23 27 28 38^ Three studies identified five barriers^30 37 38^ and one facilitator^38^ at the ‘Seeking Care’ stage (table 3, figure 2; onlinesupplemental files 58: annexures E, F, G and H).

Regarding the quality of care according to IOM domains, 15 studies identified 19 barriers,^120 22^,^30 32 36^ and 4 studies identified 10 facilitators^23 27 28 34^ to provide ‘Effective’ quality trauma care after an injury. For the ‘Timeliness’ domain, 11 studies identified 18 barriers,^124 27 29 30 32 33 35^,^38^ while 1 study described 2 facilitators^19^. Considering the provision of ‘Safe care’, 10 studies described 11 barriers,^2024 26 28 32^,^34 36^ and 3 studies identified 5 facilitators.^23 31 34^ ‘Equitable’ access to care was described in five studies identifying seven barriers,^21 22 24 35 38^ and two studies identified two facilitators.^34 38^ No studies described patient-centred care (table 3, figure 2, onlinesupplemental files 58: annexures E, F, G and H).

According to the WHO Healthcare System Building Blocks, 15 studies described ‘Healthcare service delivery’ challenges, identifying 23 barriers^121 24^,^30 32 33 35^, and 3 studies identified 4 facilitators^23 31 34^ in providing ‘Healthcare service delivery.’ Eleven studies identified seven barriers,^2022^,^24 26 28 30 32 33 36 37^ and two studies identified eight facilitators^23 34^ related to the ‘healthcare workforce’. Barriers (n=10) to providing ‘essential resources’ were described in 12 studies,^2022 24 26 28^,^30 34^ and 1 study described 2 facilitators^31^ to overcoming challenges. Only one study described ‘healthcare financing’ barriers (n=2)^22^ and two studies identified barriers (n=3)^27 34^ and facilitators (n=3)^23 34^ in ‘Healthcare governance and leadership’ (table 3, figure 2, onlinesupplemental files 58: annexures E, F, G and H).

The barriers reported at the ‘Seeking Care’ stage of the four-delay model includes self-treatment, visiting non-medical or non-trauma centres and being unable to seek timely telephonic information, especially for poisoning cases.^30 37 38^ These barriers were similarly reflected in the challenges in obtaining timely care according to the IOM quality of care domain, and some were gaps in the WHO building blocks of healthcare service delivery (online supplemental file 6: annexure F).

For the ‘Reaching Care’ stage, most barriers were around long travel time or driving distance to healthcare facilities, fragile pre-hospital services with limited geographical coverage, a lack of trained pre-hospital staff to provide patient stabilisation in the field and limited or unavailable medical supplies in ambulances.^24 26 28 30 32 36 38^ These barriers reflect gaps in pre-hospital care services, workforce and essential resources as per the WHO building blocks required to provide safe, effective, timely and equitable care as per the IOM Domains (online supplemental file 6: annexure F).

Lack of consultation with experienced physicians in the emergency room, absence of specialised trauma teams in the emergency room, insufficient sub-specialty support like neurosurgeons, cardiothoracic surgery, orthopaedics, toxicologists, interventional radiology and rehabilitation care, along with poorly established trauma care infrastructure and the absence of trauma care networks like level 1 trauma centres mainly were identified as key barriers in the ‘Receiving care’ stage.^120^,^24 30^ These barriers reflect challenges within the healthcare workforce and services at the hospital level (WHO Building Blocks) and gaps in providing effective, equitable and timely quality care (IOM Quality Domains) (online supplemental file 6: annexure F).

A few barriers were reported in the ‘Remaining in Care’ stage. These barriers were around the lack of essential resources and healthcare services (WHO Building Blocks) that prevented effective, timely and equitable care (IOM Quality Domains). These include the unavailability of spinal rehabilitation beds and patients’ low socioeconomic status, which led to an inability to get timely, expensive implants in open fracture reductions (online supplemental file 5: annexure E).^29 35^ Few studies identified barriers relevant to multiple delay stages, such as the lack of trauma registries needed to provide effective care by providing health information from reaching care to receiving care and remaining in care.^27 28^

Only six studies identified 20 facilitators,^23 27 28 31 34 38^ and establishing trauma databases/registries was the most frequently reported facilitator^23 27 28^ (table 3; online supplemental file 7: annexure G). Onlinesupplemental files 57: annexures E, F and G show all the barriers and facilitators according to the three frameworks in detail.

Eight studies reported initiatives/interventions to improve quality trauma healthcare access after injury.^123 26^,^28 31 34 38^ These included a trauma registry, trauma quality improvement initiatives, mobile-based low-cost surveillance systems, EMS interventions at multiple levels of WHO healthcare building blocks, emergency surgical and non-surgical therapeutic interventions, pre-hospital life support interventions and geospatial modelling to identify trauma care networks^123 26^,^28 31 34 38^ (online supplemental file 8:annexure G).

## Discussion

From our review of the literature, we have found that trauma care in Pakistan faces numerous challenges that undermine the health system’s ability to effectively manage injuries and provide quality care from the scene through pre-hospital, in-hospital and post-injury rehabilitation. Barriers are seen in getting care after injury across all four delay stages of seeking care, reaching care, receiving care and remaining in care. Apart from patient-centredness, barriers are described for all other IOM quality of care domains studied, including effective, safe, timely and equitable care. Although limited studies highlight challenges related to healthcare financing, governance and leadership as outlined in the WHO health system building blocks, most identified barriers pertain to healthcare service delivery, the healthcare workforce and essential resources.

Our review found that most studies were prospective, quantitative, cross-sectional epidemiological surveys conducted in urban tertiary care centres in Sindh, predominantly involving young males injured in road traffic accidents. We note that an absence of evidence does not equate to evidence that an issue does not exist, and as such, we can only highlight issues that people have attempted to study. For example, we found no studies reporting matters related to patient-centredness, likely due to this being a neglected area of research rather than patient-centred care being delivered well. Similarly, limited studies address gaps in trauma care financing, governance and leadership. We found that although a few initiatives/interventions to improve trauma care quality in Pakistan were proposed, their acceptance, feasibility or efficacy in improving access to quality trauma care is not well studied.

Nevertheless, our findings highlight substantial barriers within the trauma care system that contribute to issues in accessing quality trauma care after injury at every delay stage. The barriers identified at delay stage 1 were accessing care at non-medical facilities and being unable to get timely telephonic consultations.^37 38^ Developing a mobile-based low-cost surveillance system and a more recent initiative of empowering the community response to seek care in traumatic and lifesaving emergencies are unique initiatives for the country.^38 39^

We found that initiatives/interventions had been developed in the past decade to improve access to trauma care in delay stage 2. These initiatives/interventions reinforced public services in pre-hospital care by establishing ‘The Punjab Emergency Service Act’ of 2006, which mandates establishing and funding pre-hospital emergency services in Punjab province and sets the rules and regulations by which the service must run.^34 40^ Another initiative was establishing public-private partnerships to focus on providing essential resources and pre-hospital care training, on-job training of paramedics on curricula developed by international institutes, getting international accreditation of pre-hospital care systems, recruiting staff nurses and employing doctors as administrative staff for training and supervision, which did improve the access to trauma care after injury at delay stage 2.^34^ These initiatives emphasise the ‘Golden Hour’ concept in trauma care, which dramatically reduces the risk of death.^41^ In addition, geospatial mapping was done in Karachi, which identified the need to establish 16 trauma care centres to provide coverage during the golden hour to trauma patients in this densely populated city.^1^

However, despite these initiatives, our results have reported many barriers to improving access to quality care after injury. These include not only inadequate pre-hospital geographical coverage in the country with relatively poor coverage in rural areas but also a gap in the training of pre-hospital staff, especially in addressing time-sensitive illnesses like traumatic brain injury (TBI).^22 24 26 30 32 36^ Medical supplies are limited within the ambulances, especially a lack of oxygen tanks. This is further exacerbated due to the need for frequent ambulance maintenance owing to the poor road conditions and difficult weather.^34 36^ Thus, the care provided during pre-hospital transfers often lacks quality and timeliness and is ineffective, resulting in more complex and costly treatments and preventable mortality and morbidity.

These pre-hospital care barriers are exacerbated when health facilities are understaffed or overcrowded by patient volumes and not ready to provide timely quality care to trauma patients after injury.^42^ Our review found that issues occurring at delay stage 3 are primarily because of non-functional trauma centres and a lack of specialised trauma teams in the emergency room, including sub-specialty support.^21 22 24 25 32^ The country has no official trauma care network with defined protocols or level 1 trauma centres.^1 24 32^ Moreover, primary healthcare facilities lack the readiness to respond to injuries.^24^ This highlights the critical need for initiatives like the Amal Umer Act 2019, which seeks to improve emergency response by mandating immediate medical aid and treatment of injured persons in an emergency without waiting for medico-legal or financial formalities.^43^ It also highlights the duty of every citizen to assist injured individuals during emergencies, addressing delay stages 1 and 2.^43^

In contrast, tertiary and secondary care hospitals have inadequate advanced trauma care training and lack expertise and skills in pain management, patient safety, neuro and ENT trauma, and rehabilitation.^25^ Furthermore, a shortage of essential resources exacerbates these challenges, leading to suboptimal patient outcomes.^31^ Our review describes the scarcity of essential medical supplies, including inadequate stocks of antidotes, vaccines and resuscitation drugs.^20 24 37^ Similarly, CT scans are unavailable in many emergency rooms, which leads to a significant proportion of patients admitted without imaging.^24^ Moreover, fragmented patient care coordination within the hospital teams, lack of allied health staff, single evening operating rooms, insufficient ordering of point-of-care testing, antibiotic delays and unsafe patient referral and disposition also hamper the quality of care and increase mortality and disability.^32 33 35^ In addition, private sector hospitals often become the major trauma care providers in the country, supported by either financing from health insurance or out-of-pocket (OOP) payment for trauma care.^21 22^

We only found two studies that described facilitators as providing quality trauma care after injury at delay stages 3 & 4.^23 31^ These studies reported that establishing trauma quality improvement initiatives needs a holistic approach, starting with organisation involvement in establishing 24/7 trauma teams, nurses for post-operative care, protocols and databases.^23 27 44^ Providing 24/7 surgical and non-surgical essential resources regarding availability of resuscitation, laboratory, operating room, blood bank, radiology facilities, damage control surgeries, intervention radiology and advanced surgical equipment is required to provide timely, safe and effective trauma care.^31^ According to Hashmi *et al*, this approach is the main contributing factor to decreasing the odds of trauma-related complications and mortality by half.^23 45^ Moreover, healthcare information technology has the potential to revolutionise trauma care by improving data management, facilitating trauma registries and streamlining communications.^23 46 47^ However, several attempts at implementing trauma registries have been made in the country but have not been sustainable. Finally, the lack of an electronic medical record system makes obtaining robust data and monitoring outcomes a critical challenge.^27 28 38 44^

These findings are consistent with the studies conducted in other LMICs, where lack of awareness for seeking care and visiting non-medical facilities like traditional healers are significant barriers to seeking formal care, leading to delays and higher mortality risks, as seen in Malawi.^48^ In addition, the wider literature highlights other contributing factors, including fear, distrust of the healthcare system, financial constraints, religious beliefs, and underestimation of injury severity, which further reinforce the challenges of timely care-seeking in resource-limited settings.^48 49^ Training laypeople, like taxi drivers, in emergency first aid has proven effective in places like Madagascar and Ghana.^49 50^ In Uganda, motorcycle taxi drivers were used for hospital transport due to poor road access.^51^ Similarly, most RTA victims in India avoided ambulances due to low awareness, highlighting the need for community education campaigns and context-specific training for adequate emergency care access.^51^ Moreover, training bystander response in time-sensitive illnesses improved pre-hospital patient quality outcomes similar to our findings.^22 26 52^

Delays in stage 2 ‘reaching care’ are more pronounced in LMICs compared with resource-rich settings, mainly due to limited pre-hospital care capacity and high rates of traumatic brain injury (TBI) from road traffic injuries.^50^ The pre-hospital care system in LMICs is fragmented and uncoordinated like Pakistan, lacking trained medical personnel and first responders, inadequate basic materials and substandard infrastructure.^53^ For example, a study done on TBI patients in Cambodia found that increasing injury to admission time was associated with worsening outcomes, highlighting the need for prehospital guidelines for TBI care in LMICs.^54 55^

Infrastructure improvement, enhanced education and training for trauma care providers and developing and implementing trauma registries are crucial steps towards building more robust trauma quality care improvement interventions in LMICs.^945^,^47 56 57^ Training programmes like the WHO’s Trauma Care Checklist, the ATLS (Advanced Trauma Life Support) and primary trauma care courses (PTC) have been proven to increase the capacity of trauma systems in resource-limited settings.^58^,^62^

In addition, barriers to implementing a trauma registry in our country are like those in other LMICs, which require electronic health records, training personnel, operational costs and human resources funding.^27 46 47^ In many LMICs, while surgical costs are often subsidised, patients face significant financial and logistical barriers to accessing essential diagnostics like CT scans. In Kampala, Uganda, the lack of a functional CT scanner at the tertiary hospital forces patients to travel to private facilities and pay $80–$130. These challenges hinder access to critical interventions and exacerbate health inequities.^50^

Delay in stage 4, in Sub-Saharan Africa, 210 million people need rehabilitation services, but access is extremely limited due to shortages of psychiatrists and occupational therapists, scarce facilities and low availability of therapies and assistive devices, especially in rural areas.^63^ Over half of Anglophone countries lack physiotherapy and occupational therapy programmes, with advanced training available only in South Africa and Nigeria. Strengthening rehabilitation services and training is crucial to addressing these gaps.^63^

Moreover, many of the identified barriers, such as limited prehospital services, lack of trained specialised workforce, lack of essential resources and registries, are not unique to providing quality trauma care but also affect patients with other time-sensitive illnesses like cardiac arrest, stroke, neonatal and obstetric emergencies and sepsis.^64^,^69^ Therefore, strengthening the quality of trauma care within Pakistan’s health system should not be an isolation; rather, the need offers an opportunity to reform the quality of the healthcare system broadly, especially for acute care.^10^ Similar challenges to what we have found have been documented in nearby India, where inadequate pre-hospital care and communication, frequent medical errors, delays in transfusion and imaging and limited surgical capacity hinder trauma resuscitation^[70-72]^. Therefore, this health systems-oriented approach is consistent with global calls to integrate quality trauma care into broader emergency and acute care reform in low- and middle-income countries.^12^

The major limitation of our review was the date and language restriction; we only included studies published in English and within the last 10 years, which might have led to missing evidence beyond this criteria. In addition, the evidence from the studies was low. Most studies were epidemiological cross-sectional data published primarily in locally indexed trauma and injury care journals. Only a few studies holistically address the challenges in trauma care systems and the quality of care the IOM domain provides. There is no literature addressing patient-centredness. Similarly, we were unable to find studies addressing the health economic burden of trauma care morbidities and mortalities. There are no interventional studies or randomised control trials in trauma care after injury in the country. Another limitation is that the included studies were of variable quality, as identified by the CASP assessments. While we retained all studies due to the scarcity of literature, the methodological weaknesses of some studies may affect the robustness of the overall findings.

## Conclusion

Our findings of barriers, facilitators and current intervention status align with the need for developing feasible, sustainable, contextually relevant interventions to improve access to quality trauma care after injury in Pakistan. This synthesis highlighted the scarcity of available literature, identified gaps and informed the trauma care experts to take one step forward, engage stakeholders in prioritising these barriers and facilitators and co-design potential solutions above and beyond existing initiatives/interventions. This will ensure the intervention’s sustainability and pave the way for localised, scalable, contextually relevant and feasible solutions beyond existing solutions, particularly for the young male population, and reduce the economic burden on the overall healthcare system.

Simultaneously, our finding also indicates that strengthening the existing Pakistan trauma care workforce, infrastructure, processes and essential resources aligned with the WHO healthcare system is required to provide effective, safe and timely care, in accordance with the IoM quality domains, at each stage of the trauma care system to improve access to quality care in the country.

## Supplementary material

10.1136/bmjopen-2025-101071 online supplemental file 1

10.1136/bmjopen-2025-101071 online supplemental file 2

10.1136/bmjopen-2025-101071 online supplemental file 3

10.1136/bmjopen-2025-101071 online supplemental file 4

10.1136/bmjopen-2025-101071 online supplemental file 5

10.1136/bmjopen-2025-101071 online supplemental file 6

10.1136/bmjopen-2025-101071 online supplemental file 7

10.1136/bmjopen-2025-101071 online supplemental file 8

## Data Availability

All data relevant to the study are included in the article or uploaded as supplementary information.
