# Supplementary material for: Access to quality trauma care after injury in Pakistan: a systematic review and narrative synthesis
Source: BMJ Open. 2025 Dec 7;15(12):e101071. doi: 10.1136/bmjopen-2025-101071 (PMC12699741; doi:10.1136/bmjopen-2025-101071)
Supplement: online supplemental file 1 [file bmjopen-15-12-s001.docx]

**Supplementary File 1**

**Annexure A: Search Database and Search Strategy**

**MEDLINE**

| # | Query |  |
| --- | --- | --- |
| 1 | ("Road traffic accidents" or "Falls" or "Drowning" or "Burns" or "Poisoning" or "Violence" or "Physical Abuses" or "Firearm" or "Gunshot Injuries" or "Wounds" or "Trauma" or "Injuries" or "Polytrauma").mp. [mp=title, book title, abstract, original title, name of substance word, subject heading word, floating sub-heading word, keyword heading word, organism supplementary concept word, protocol supplementary concept word, rare disease supplementary concept word, unique identifier, synonyms, population supplementary concept word, anatomy supplementary concept word] | 1,233,811 |
| 2 | ("Emergency Health Services" or "Pre-Hospital Care" or "Hospital-based Facilities" or "Trauma Centers" or "Trauma Center Network" or "Emergency Departments" or "Emergency Room" or "Emergency medical services" or "Accident Scenes" or "Rehabilitation" or "Aftercare").mp. [mp=title, book title, abstract, original title, name of substance word, subject heading word, floating sub-heading word, keyword heading word, organism supplementary concept word, protocol supplementary concept word, rare disease supplementary concept word, unique identifier, synonyms, population supplementary concept word, anatomy supplementary concept word] | 455,681 |
| 3 | ("quality trauma care" or "quality of care" or " Delay in care" or "Access" or "Institute of Medicine" or "IOM" or "WHO health system framework" or "Trauma care system" or "Integrated Trauma Care" or "Safety" or "Equitable" or "Equity" or "Timely" or "Mortality" or "Morbidity" or "Disability*" or "Survival").mp. [mp=title, book title, abstract, original title, name of substance word, subject heading word, floating sub-heading word, keyword heading word, organism supplementary concept word, protocol supplementary concept word, rare disease supplementary concept word, unique identifier, synonyms, population supplementary concept word, anatomy supplementary concept word] | 3,599,708 |
| 4 | Pakistan/ | 23,367 |
| 5 | 1 and 2 and 3 and 4 | 59 |
| 6 | limit 5 to yr="2013 - 2023" | 40 |
| 7 | Animals/ not (Animals/ and Humans/) | 5,166,308 |
| 8 | (("animal*" or "Cat*" or "Dog*" or "feline*" or "hamster*" or "mice*" or "monkey*" or "Mouse*" or "Murine*" or "Pig*" or "Porcine*" or "primate*" or "rabbit*" or "rat*" or "rodent*" or "sheep*") not (human* or patient*)).mp. [mp=title, book title, abstract, original title, name of substance word, subject heading word, floating sub-heading word, keyword heading word, organism supplementary concept word, protocol supplementary concept word, rare disease supplementary concept word, unique identifier, synonyms, population supplementary concept word, anatomy supplementary concept word] | 5,659,371 |
| 9 | 6 not (7 or 8) | 40 |
| 10 | remove duplicates from 9 | 40 |
| 11 | ("quality trauma care" or "quality of care" or " Delay in care" or "Access" or "Institute of Medicine" or "IOM" or "WHO health system framework" or "Trauma care system" or "Integrated Trauma Care" or "Safety" or "Equitable" or "Equity" or "Timely" or "Mortality" or "Morbidity" or "Disability*" or "Survival analysis" or "health personnel" or "health administration" or "universal health care" or "delivery of healthcare" or " health information system" or "healthcare resources" or "healthcare disparity" or "trauma registries" or "trauma quality surveys" or "quality of healthcare").mp. [mp=title, book title, abstract, original title, name of substance word, subject heading word, floating sub-heading word, keyword heading word, organism supplementary concept word, protocol supplementary concept word, rare disease supplementary concept word, unique identifier, synonyms, population supplementary concept word, anatomy supplementary concept word] | 2,972,088 |
| 12 | 5 and 11 | 56 |
| 13 | 12 not (7 or 8) | 56 |
| 14 | remove duplicates from 13 | 56 |
| 15 | limit 14 to yr="2013 - 2023" | 39 |

**Embase**

| # | Query |  |
| --- | --- | --- |
| 1 | ("Road traffic accidents" or "Falls" or "Drowning" or "Burns" or "Poisoning" or "Violence" or "Physical Abuses" or "Firearm" or "Gunshot Injuries" or "Wounds" or "Trauma" or "Injuries" or "Polytrauma").mp. [mp=title, abstract, heading word, drug trade name, original title, device manufacturer, drug manufacturer, device trade name, keyword heading word, floating subheading word, candidate term word] | 1,064,547 |
| 2 | ("Emergency Health Services" or "Pre-Hospital Care" or "Hospital-based Facilities" or "Trauma Centers" or "Trauma Center Network" or "Emergency Departments" or "Emergency Room" or "Emergency medical services" or "Accident Scenes" or "Rehabilitation" or "Aftercare").mp. [mp=title, abstract, heading word, drug trade name, original title, device manufacturer, drug manufacturer, device trade name, keyword heading word, floating subheading word, candidate term word] | 557,762 |
| 3 | ("quality trauma care" or "quality of care" or " Delay in care" or "Access" or "Institute of Medicine" or "IOM" or "WHO health system framework" or "Trauma care system" or "Integrated Trauma Care" or "Safety" or "Equitable" or "Equity" or "Timely" or "Mortality" or "Morbidity" or "Disability*" or "Survival analysis" or "health personnel" or "health administration" or "universal health care" or "delivery of healthcare" or " health information system" or "healthcare resources" or "healthcare disparity" or "trauma registries" or "trauma quality surveys" or "quality of healthcare").mp. [mp=title, abstract, heading word, drug trade name, original title, device manufacturer, drug manufacturer, device trade name, keyword heading word, floating subheading word, candidate term word] | 4,974,818 |
| 4 | Pakistan/ | 38,754 |
| 5 | 1 and 2 and 3 and 4 | 71 |
| 6 | Animals/ not (Animals/ and Humans/) | 1,091,741 |
| 7 | (("animal*" or "Cat*" or "Dog*" or "feline*" or "hamster*" or "mice*" or "monkey*" or "Mouse*" or "Murine*" or "Pig*" or "Porcine*" or "primate*" or "rabbit*" or "rat*" or "rodent*" or "sheep*") not (human* or patient*)).mp. [mp=title, abstract, heading word, drug trade name, original title, device manufacturer, drug manufacturer, device trade name, keyword heading word, floating subheading word, candidate term word] | 6,792,944 |
| 8 | 5 not (6 or 7) | 70 |
| 9 | remove duplicates from 8 | 69 |
| 10 | limit 9 to yr="2013 - 2023" | 48 |

**Web of Science**

(TS=(("Road traffic accidents" or "Falls" or "Drowning" or "Burns" or "Poisoning" or "Violence" or "Physical Abuses" or "Firearm" or "Gunshot Injuries" or "Wounds" or "Trauma" or "Injuries" or "Polytrauma").) AND TS=(("Emergency Health Services" or "Pre-Hospital Care" or "Hospital-based Facilities" or "Trauma Centers" or "Trauma Center Network" or "Emergency Departments" or "Emergency Room" or "Emergency medical services" or "Accident Scenes" or "Rehabilitation" or "Aftercare").) AND TS=(("quality trauma care" or "quality of care" or " Delay in care" or "Access" or "Institute of Medicine" or "IOM" or "WHO health system framework" or "Trauma care system" or "Integrated Trauma Care" or "Safety" or "Equitable" or "Equity" or "Timely" or "Mortality" or "Morbidity" or "Disability*" or "Survival").) AND TS=(Pakistan) NOT TS=((("animal*" or "Cat*" or "Dog*" or "feline*" or "hamster*" or "mice*" or "monkey*" or "Mouse*" or "Murine*" or "Pig*" or "Porcine*" or "primate*" or "rabbit*" or "rat*" or "rodent*" or "sheep*") not (human* or patient*)).)) AND ((PY==("2023" OR "2022" OR "2021" OR "2020" OR "2019" OR "2018" OR "2017" OR "2016" OR "2014" OR "2013" OR "2015")) NOT (SILOID==("PPRN")))

**Scopus**

(TITLE-ABS-KEY(( "Road traffic accidents" OR "Falls" OR "Drowning" OR "Burns" OR "Poisoning" OR "Violence" OR "Physical Abuses" OR "Firearm" OR "Gunshot Injuries" OR "Wounds" OR "Trauma" OR "Injuries" OR "Polytrauma" )) AND TITLE-ABS-KEY(( "Emergency Health Services" OR "Pre-Hospital Care" OR "Hospital-based Facilities" OR "Trauma Centers" OR "Trauma Center Network" OR "Emergency Departments" OR "Emergency Room" OR "Emergency medical services" OR "Accident Scenes" OR "Rehabilitation" OR "Aftercare" )) AND TITLE-ABS-KEY(( "quality trauma care" OR "quality of care" OR " Delay in care" OR "Access" OR "Institute of Medicine" OR "IOM" OR "WHO health system framework" OR "Trauma care system" OR "Integrated Trauma Care" OR "Safety" OR "Equitable" OR "Equity" OR "Timely" OR "Mortality" OR "Morbidity" OR "Disability*" OR "Survival analysis" OR "health personnel" OR "health administration" OR "universal health care" OR "delivery of healthcare" OR " health information system" OR "healthcare resources" OR "healthcare disparity" OR "trauma registries" OR "trauma quality surveys" OR "quality of healthcare" ) .) AND TITLE-ABS-KEY(pakistan) AND NOT TITLE-ABS-KEY(( "animal*" OR "Cat*" OR "Dog*" OR "feline*" OR "hamster*" OR "mice*" OR "monkey*" OR "Mouse*" OR "Murine*" OR "Pig*" OR "Porcine*" OR "primate*" OR "rabbit*" OR "rat*" OR "rodent*" OR "sheep*" ))) AND PUBYEAR > 2012 AND PUBYEAR < 2024

**Cochrane**

0 Cochrane Reviews matching ("Road traffic accidents" or "Falls" or "Drowning" or "Burns" or "Poisoning" or "Violence" or "Physical Abuses" or "Firearm" or "Gunshot Injuries" or "Wounds" or "Trauma" or "Injuries" or "Polytrauma") in Title Abstract Keyword AND ("Emergency Health Services" or "Pre-Hospital Care" or "Hospital-based Facilities" or "Trauma Centers" or "Trauma Center Network" or "Emergency Departments" or "Emergency Room" or "Emergency medical services" or "Accident Scenes" or "Rehabilitation" or "Aftercare"). in Title Abstract Keyword AND ("quality NEXT trauma NEXT care" or "quality" or "Delay or Access" or "Institute NEXT of NEXT Medicine" or "WHO NEXT health NEXT system NEXT framework" or "Trauma NEXT care NEXT system" or "Integrated NEXT Trauma NEXT Care" or "Safety" or "Equitable" or "Equity" or "Timely" or "Mortality" or "Morbidity" or "Disability" or "Survival") in Title Abstract Keyword AND Pakistan in Title Abstract Keyword - (Word variations have been searched)

**Proquest**

(noft(("Road traffic accidents" OR "Falls" OR "Drowning" OR "Burns" OR "Poisoning" OR "violences" OR "Physical Abuses" OR "Firearm" OR "Gunshot Injuries" OR "Wounds" OR "Trauma" OR "Injuries" OR "Polytrauma")) AND noft(("Emergency Health Services" OR "Pre-Hospital Care" OR "Hospital-based Facilities" OR "Trauma Centers" OR "Trauma Center Network" OR "Emergency Departments" OR "Emergency Room" OR "Emergency medical services" OR "Accident Scenes" OR "Rehabilitation" OR "Aftercare")) AND noft(("quality trauma care" OR "quality of care" OR " Delay in care" OR "Access" OR "Institute of Medicine" OR "IOM" OR "WHO health system framework" OR "Trauma care system" OR "Integrated Trauma Care" OR "Safety" OR "Equitable" OR "Equity" OR "Timely" OR "Mortality" OR "Morbidity" OR "Disability*" OR "Survival analysis" OR "health personnel" OR "health administration" OR "universal health care" OR "delivery of healthcare" OR " health information system" OR "healthcare resources" OR "healthcare disparity" OR "trauma registries" OR "trauma quality surveys" OR "quality of healthcare")) AND noft(Pakistan) NOT noft(Animals/ NOT (Animals/ AND Humans/)) NOT noft((("animal*" OR "Cat*" OR "Dog*" OR "feline*" OR "hamster*" OR "mice*" OR "monkey*" OR "Mouse*" OR "Murine*" OR "Pig*" OR "Porcine*" OR "primate*" OR "rabbit*" OR "rat*" OR "rodent*" OR "sheep*") NOT (human* OR patient*)) .)) AND pd(20130101-20231231)
