# Supplementary material for: Access to quality trauma care after injury in Pakistan: a systematic review and narrative synthesis
Source: BMJ Open. 2025 Dec 7;15(12):e101071. doi: 10.1136/bmjopen-2025-101071 (PMC12699741; doi:10.1136/bmjopen-2025-101071)
Supplement: online supplemental file 2 [file bmjopen-15-12-s002.DOC]

**Supplementary File 2**

**Annexure B: Studies Quality Assessment : Critical Appraisal Skills Programme (CASP) Quality Appraisal Tool Scores**

| **Critical Appraisal Skills Programme (CASP) Quality appraisal tool scores** | | | | | | | | | | | | |
| --- | --- | --- | --- | --- | --- | --- | --- | --- | --- | --- | --- | --- |
| **Yes (2)/Can’t Tell (1)/ No (0)** | | | | | | | | | | | | |
| **Cross-sectional Studies** | **Did the study address a clearly focused issue?** | **Did the authors use an appropriate method to answer their question?** | **Were the subjects recruited in an acceptable way?** | **Were the measures accurately measured to reduce bias?** | **Were the data collected in a way that addressed the research issue?** | **Did the study have enough participants to minimise the play of chance?** | **How are the results presented and what is the main result?** | **Was the data analysis sufficiently rigorous?** | **Is there a clear statement of findings?** | **Can the results be applied to the local population?** | **How valuable is the research?** | **Total Score (22 points)** |
| **New et al. 2013** | 2 | 2 | 2 | 1 | 2 | 0 | 2 | 0 | 2 | 2 | 2 | 17 |
| **Mehmood et al.  2013** | 2 | 2 | 2 | 2 | 2 | 2 | 2 | 2 | 2 | 1 | 2 | 21 |
| **Hashmi et al.  2013** | 2 | 2 | 2 | 2 | 2 | 2 | 2 | 2 | 2 | 1 | 2 | 21 |
| **Bhatti et al 2013** | 2 | 2 | 2 | 2 | 2 | 2 | 2 | 2 | 2 | 0 | 0 | 18 |
| **Zaidi et al.  2013** | 2 | 2 | 2 | 2 | 2 | 2 | 2 | 2 | 2 | 2 | 2 | 22 |
| **Khan et al. 2014** | 2 | 2 | 2 | 1 | 2 | 2 | 2 | 0 | 2 | 2 | 2 | 19 |
| **Bhatti et al. 2015** | 2 | 2 | 2 | 2 | 2 | 2 | 2 | 2 | 2 | 2 | 2 | 22 |
| **Arsalan**  **et.al  2016** | 2 | 2 | 1 | 2 | 1 | 0 | 2 | 2 | 0 | 2 | 0 | 14 |
| **Minhas et al. 2017** | 2 | 1 | 2 | 0 | 1 | 0 | 2 | 0 | 2 | 2 | 2 | 14 |
| **Rizwan et al.  2018** | 2 | 2 | 2 | 1 | 2 | 1 | 2 | 0 | 2 | 2 | 2 | 18 |
| **Salman et al.  2020** | 2 | 2 | 2 | 2 | 2 | 2 | 2 | 2 | 2 | 1 | 2 | 21 |
| **Saqib et al.  2020** | 2 | 2 | 2 | 1 | 2 | 1 | 2 | 2 | 2 | 1 | 2 | 19 |
| **Ihsan et al. 2020** | 2 | 2 | 0 | 0 | 2 | 0 | 2 | 0 | 2 | 2 | 2 | 14 |
| **Khalil et al. 2021** | 2 | 2 | 0 | 2 | 2 | 0 | 2 | 2 | 2 | 2 | 2 | 18 |
| **Ashraf et al.  2022** | 2 | 2 | 2 | 1 | 2 | 2 | 2 | 2 | 2 | 2 | 2 | 21 |
| **Rahman et al 2022** | 2 | 2 | 1 | 2 | 2 | 1 | 2 | 2 | 2 | 0 | 0 | 16 |
| **Bakhshi et al.  2023** | 2 | 2 | 2 | 1 | 2 | 2 | 2 | 2 | 2 | 2 | 2 | 21 |

| **Critical Appraisal Skills Programme (CASP) Quality appraisal tool scores** | | | | | | | | | | | | | | |
| --- | --- | --- | --- | --- | --- | --- | --- | --- | --- | --- | --- | --- | --- | --- |
| **Yes (2)/Can’t Tell (1)/ No (0)** | | | | | | | | | | | | | | |
| **Cohort  Studies** | **Did the study address a clearly focused issue?** | **Was the cohort recruited in an acceptable way** | **Was the exposure accurately measured to minimise bias?** | **Was the outcome accurately measured to minimise bias?** | **Have the authors identified all important confounding factors?** | **Have they take account of the confounding factors in the design and/or analysis?** | **Was the follow up of subjects complete enough?** | **Was the follow up of subjects long enough?** | **How precise are the results?** | **Do you believe the results?** | **Can the results be applied to the local population?** | **Do the results of this study fit with other available evidence?** | **What are the implications of this study for practice?** | **Total Score**  **(26 points)** |
| **Mawani et al. 2018** | 2 | 2 | 2 | 2 | 2 | 1 | 1 | 1 | 2 | 2 | 2 | 2 | 2 | 23 |
| **Tahir et al.  2021** | 2 | 2 | 2 | 2 | 2 | 2 | 2 | 2 | 2 | 2 | 2 | 2 | 2 | 26 |

| **Critical Appraisal Skills Programme (CASP) Quality appraisal tool scores** | | | | | | | | | | | |
| --- | --- | --- | --- | --- | --- | --- | --- | --- | --- | --- | --- |
| **Yes (2)/Can’t Tell (1)/ No (0)** | | | | | | | | | | | |
| **Qualitative Study** | **Was there a clear statement of the aims of the research?** | **Is a qualitative methodology appropriate?** | **Was the research design appropriate to address the aims of the research?** | **Was the recruitment strategy appropriate to the aims of the research?** | **Was the data collected in a way that addressed the research issue?** | **Has the relationship between researcher and participants been adequately considered?** | **Have ethical issues been taken into consideration?** | **Was the data analysis sufficiently rigorous?** | **Is there a clear statement of findings?** | **How valuable is the research?** | **Total Number (20 points)** |
| **Sriram et al. 2024** | 2 | 2 | 2 | 2 | 2 | 0 | 2 | 2 | 2 | 2 | 18 |
