# Supplementary material for: Access to quality trauma care after injury in Pakistan: a systematic review and narrative synthesis
Source: BMJ Open. 2025 Dec 7;15(12):e101071. doi: 10.1136/bmjopen-2025-101071 (PMC12699741; doi:10.1136/bmjopen-2025-101071)
Supplement: online supplemental file 3 [file bmjopen-15-12-s003.docx]

| **S. No** | **First Author & Year Published** | **Quantitative/ Qualitative** | **Study Design** | **Prospective/ Retrospective** | **Study Duration** | **Study Site** | **Geographical Location (Urban**  **/Rural)** | **Study Settings** | **Population** | **Data collection methods** | **Patient related studies & Facilities related studies** | **Sample size** | **Mean Age** | **Gender** | **Mechanism of Injury** | **Injury Type** | **Intervention Delivered (Yes/No)** | **Quality Assessment** |
| --- | --- | --- | --- | --- | --- | --- | --- | --- | --- | --- | --- | --- | --- | --- | --- | --- | --- | --- |
| 1 | Mehmood et al.  2013 | Quantitative | Cross-sectional | Prospective | 3 months | Karachi, Sindh | Urban | Tertiary Care | All Age Groups | Patient Medical Records | Patient related studies | 542 trauma patients | 27 years | Male (72.6%) | Fall | Head, Face & upper extremities | Yes | 21/22 |
| 2 | New et al. 2013 | Quantitative | Cross-sectional | Prospective | 35 months | Rawalpindi | Urban | Tertiary care | Adults | Structured questionnaire | Facility related studies | NA | NA | NA | NA | Spinal Cord Injury | No | 17/22 |
| 3 | Hashmi et al. 2013 | Quantitative | Cross-sectional | Retrospective & Prospective | 12 months | Karachi, Sindh | Urban | Tertiary Care | All Age Groups | Trauma Registry | Patient related studies | 1227 trauma patients | NA | Male (86%) | RTA | Head & Neck Injuries | Yes | 21/22 |
| 4 | Bhatti et al. 2013 | Quantitative | Cross-sectional | Prospective | NA | Sindh | NA | Pre-hospital Care | Ambulance Staff | Structured questionnaire | Facility related studies | 14 Ambulance stations, 12 Ambulances & 13 Ambulance staff | NA | NA | NA | NA | No | 18/22 |
| 5 | Zaidi et al. 2013 | Quantitative | Cross-sectional | Prospective | 24 months | Pakistan | Urban & Rural | Tertiary Care | All Age Groups | Structured questionnaire | Patient related studies | 6212 dog-bites patients | 20 years | Male (79.4%) | Dog-Bite | NA | Yes | 22/22 |
| 6 | Khan et al. 2014 | Quantitative | Cross-sectional | Prospective | 6 months | Karachi, Faisalabad | Urban | Tertiary Care | NA | Structured questionnaire | Facility related studies | 2 poison control centers | NA | NA | NA | NA | No | 19/22 |
| 7 | Bhatti et al. 2015 | Quantitative | Cross-sectional | Prospective | 4 months | Karachi, Lahore, Islamabad, Quetta, Rawalpindi & Peshawar | Urban | Tertiary Care | All Age Groups | Structured questionnaire | Patient related studies | 803 TBI patients | 25-44 years | Male (76%) | RTA | Head Injury - TBI | No | 22/22 |
| 8 | Arslan et al. 2016 | Quantitative | Cross-sectional | Prospective | NA | Punjab | Urban | Tertiary Care | Emergency Physician | Structured questionnaire | Facility related studies | 17 facilities | NA | NA | Poisoning | NA | No | 14/22 |
| 9 | Sriram et al. 2016 | Qualitative | NA | NA | 1 month | Pakistan | Urban | Pre-hospital Care | NA | IDIs, Document review & non-participant observation | Facility related studies | 3 organizations | NA | NA | NA | NA | Yes | 18/20 |
| 10 | Minhas et al. 2017 | Quantitative | Cross-sectional | Prospective | 6 months | Karachi, Sindh | Urban | Tertiary care | All Age Groups | Trauma Registry | Patient related studies | 1500 trauma patients | 45 years | Male (68.7%) | RTA | Femur Fractures | Yes | 14/22 |
| 11 | Rizwan et al. 2018 | Quantitative | Cross-sectional | Retrospective | 6 months | Karachi, Sindh | Urban | Tertiary Care | All Age Groups | Patient medical records | Patient related studies | 32 trauma patients | 36 years | Male (87.5%) | Gunshot | Penetrating Injuries | Yes | 18/22 |
| 12 | Mawani et al. 2018 | Quantitative | Cohort Study | Prospective | 4 months | Karachi, Sindh | Urban | Pre-Hospital Care & Tertiary Care | Adults | Structured questionnaire | Patient related studies | 187 trauma patients | 35.1 years | Male (95.2%) | Gunshot | Penetrating Injuries | Yes | 23/26 |
| 13 | Ihsan et al. 2020 | Quantitative | Cross-sectional | Prospective | 18 months | Punjab | Urban & Rural | Tertiary Care Hospital, Trauma Center, Secondary Care Hospital (DHQ) | All Age Groups | Structured questionnaire | Facility related studies | 36 districts | 11-40 years | Male (79%) | RTA | Head Injuries | No | 14/22 |
| 14 | Salman et al. 2020 | Quantitative | Cross-sectional | Retrospective | 1 year | Karachi, Sindh | Urban | Tertiary Care | All Age Groups | Patient medical records | Patient related studies | 972 trauma patients | 36 years | Male (68.2%) | RTA | MSK Injuries  Head & Neck Injuries | No | 21/22 |
| 15 | Saqib et al. 2020 | Quantitative | Cross-sectional | Retrospective | 12 months | Karachi, Sindh | Urban | Tertiary Care | Adults | Patient medical records | Patient related studies | 146 trauma patients | 37.4 years | Male (82.2%) | RTA | NA | No | 19/22 |
| 16 | Khalil et al. 2021 | Quantitative | Cross-sectional | Prospective | 4 months | Karachi, Sindh | Urban | Tertiary care & Secondary care | Emergency Physician | Structured questionnaire | Facility related studies | 12 facilities | NA | NA | NA | NA | No | 19/22 |
| 17 | Tahir et al. 2021 | Quantitative | Cohort Study | Prospective | 35 weeks | Karachi, Sindh | Urban | Tertiary Care | Adults | Structured questionnaire | Patient related studies | 1896 trauma patients | 43.52 years | Male (72.2%) | RTA | Open tibial fractures | No | 26/26 |
| 18 | Ashraf et al. 2022 | Quantitative | Cross-sectional | Prospective | NA | Karachi, Sindh | Urban | Tertiary Care & Secondary Care | NA | Facility Registry, Institution Websites & Online Sources | Facility related studies | 54 facilities | NA | NA | NA | NA | Yes | 21/22 |
| 19 | Rahman et al. 2022 | Quantitative | Cross-sectional | Prospective | 3 weeks | Multan - Southern Punjab | Urban | Tertiary Care | All Age Groups | Structured questionnaire | Patient related studies | 468 TBI patients | 28.9  years | Male (84.2%) | RTA | Traumatic Brain Injury | No | 16/22 |
| 20 | Bakhshi et al. 2023 | Quantitative | Cross-sectional | Prospective | 8 months | Pakistan | Urban & Rural | Tertiary Care | Neurosurgeons | Structured questionnaire | Facility related studies | 307 neurosurgeons & 74 neurosurgery centers | NA | NA | NA | NA | No | 21/22 |
