# Supplementary material for: Access to quality trauma care after injury in Pakistan: a systematic review and narrative synthesis
Source: BMJ Open. 2025 Dec 7;15(12):e101071. doi: 10.1136/bmjopen-2025-101071 (PMC12699741; doi:10.1136/bmjopen-2025-101071)
Supplement: online supplemental file 4 [file bmjopen-15-12-s004.docx]

**Supplementary File 4: Annexure D: Summary of Study Population Across Included Studies**

| Variables (n= number of studies reported) | Cumulative Total (n) | Mean/Range/ Percentage |
| --- | --- | --- |
| Total participants (n=11) | 15,060 | 32-6,212 |
| Age (years) (n=9)* | 14,922 | 34.5 years (1-89 years) |
| Gender (n=11) | 14,922 | Male – 11,628 (77.9%)  Female – 3,294 (22.07%) |
| Education | NA | Not reported in any study |
| Socioeconomic status | NA | Not reported in any study |

*9 studies reported mean ages.
