# Supplementary material for: Access to quality trauma care after injury in Pakistan: a systematic review and narrative synthesis
Source: BMJ Open. 2025 Dec 7;15(12):e101071. doi: 10.1136/bmjopen-2025-101071 (PMC12699741; doi:10.1136/bmjopen-2025-101071)
Supplement: online supplemental file 5 [file bmjopen-15-12-s005.docx]

**Supplementary File 4**

**Annexure D: Mapping of Studies on Four Delay Model, Who Healthsystem Building Blocks & Institute of Medicine (IoM) Quality Domains**

|  | **Four Dealy Model (D)** | | | | **WHO Healthsystem Building Blocks (HS)** | | | | | | **Institute of Medicine (IOM) Quality Domain (Q)** | | | | | **Studies Identifying Delays, Barriers, Facilitators & Challenges in Improving Access to Quality Trauma Care After Injury In Pakistan** |
| --- | --- | --- | --- | --- | --- | --- | --- | --- | --- | --- | --- | --- | --- | --- | --- | --- |
| **First Author Year Published** | **Seeking Care** | **Reaching Care** | **Receiving Care** | **Remaining In Care** | **Healthcare Services** | **Healthcare Workforce** | **Essential Resources** | **Healthcare Information Technology** | **Healthcare finances** | **Healthcare Governance & Leadership** | **Safe** | **Effective** | **Timely** | **Equitable** | **Patient -Centered** |  |
| **Mehmood et al.  2013** |  | **✓** | **✓** | **✓** | **✓** |  |  | **✓** |  | **✓** |  | **✓** | **✓** |  |  | **D, HS, Q** |
| **New et al. 2013** |  |  |  | **✓** | **✓** |  | **✓** |  |  |  |  | **✓** | **✓** |  |  | **D, HS, Q** |
| **Hashmi et al. 2013** |  |  | **✓** | **✓** | **✓** | **✓** | **✓** | **✓** |  |  | **✓** | **✓** | **✓** |  |  | **D, HS, Q** |
| **Bhatti et al. 2013** |  | **✓** |  |  | **✓** | **✓** | **✓** |  |  |  | **✓** | **✓** | **✓** |  |  | **D, HS, Q** |
| **Zaidi et al. 2013** | **✓** | **✓** | **✓** | **✓** | **✓** |  | **✓** | **✓** |  |  | **✓** | **✓** | **✓** | **✓** |  | **D, HS, Q** |
| **Khan et al. 2014** | **✓** |  | **✓** |  | **✓** | **✓** | **✓** | **✓** |  |  | **✓** | **✓** | **✓** |  |  | **D, HS,Q** |
| **Bhatti et al. 2015** |  | **✓** | **✓** | **✓** | **✓** | **✓** | **✓** |  | **✓** |  | **✓** | **✓** |  | **✓** |  | **D,HS,Q** |
| **Arslan et al. 2016** |  |  | **✓** |  |  | **✓** | **✓** |  |  |  | **✓** | **✓** |  |  |  | **D,HS,Q** |
| **Sriram et al. 2016** |  | **✓** |  |  | **✓** | **✓** | **✓** |  |  | **✓** | **✓** | **✓** |  | **✓** |  | **D,HS,Q** |
| **Minhas et al. 2017** |  | **✓** | **✓** | **✓** | **✓** | **✓** | **✓** | **✓** |  |  | **✓** | **✓** |  |  |  | **D,HS,Q** |
| **Rizwan et al. 2018** |  |  | **✓** |  | **✓** |  | **✓** |  |  |  | **✓** |  |  |  |  | **D,HS,Q** |
| **Mawani et al. 2018** |  | **✓** | **✓** |  | **✓** | **✓** | **✓** |  |  |  | **✓** | **✓** |  |  |  | **D, HS, Q** |
| **Ihsan et al. 2020** |  | **✓** | **✓** |  | **✓** | **✓** | **✓** |  |  |  | **✓** | **✓** | **✓** | **✓** |  | **D, HS, Q** |
| **Salman et al. 2020** |  | **✓** | **✓** |  | **✓** | **✓** | **✓** |  |  |  | **✓** | **✓** | **✓** |  |  | **D,HS, Q** |
| **Saqib et al. 2020** |  |  | **✓** |  | **✓** | **✓** | **✓** |  |  |  | **✓** |  | **✓** |  |  | **D,HS, Q** |
| **Khalil et al. 2021** |  |  | **✓** |  | **✓** | **✓** | **✓** |  |  |  | **✓** | **✓** |  |  |  | **D, HS, Q** |
| **Tahir et al. 2021** |  |  | **✓** | **✓** | **✓** |  | **✓** |  |  |  | **✓** |  | **✓** | **✓** |  | **D, HS, Q** |
| **Ashraf et al. 2022** |  | **✓** | **✓** |  | **✓** |  |  |  |  |  | **✓** | **✓** | **✓** |  |  | **D, HS, Q** |
| **Rahman et al. 2022** | **✓** | **✓** | **✓** |  | **✓** | **✓** | **✓** |  |  |  | **✓** | **✓** | **✓** |  |  | **D, HS, Q** |
| **Bakhshi et al. 2023** |  |  | **✓** |  | **✓** | **✓** |  |  |  |  |  |  |  | **✓** |  | **D, HS, Q** |
