# Supplementary material for: Access to quality trauma care after injury in Pakistan: a systematic review and narrative synthesis
Source: BMJ Open. 2025 Dec 7;15(12):e101071. doi: 10.1136/bmjopen-2025-101071 (PMC12699741; doi:10.1136/bmjopen-2025-101071)
Supplement: online supplemental file 6 [file bmjopen-15-12-s006.docx]

**Annexure E:**

**Issues found affecting access to Quality Trauma Care After Injury In Pakistan described as Barriers & Facilitators and categorized using the four Delay, IoM, and WHO building blocks**

**Systematic Review & Narrative Synthesis Results – Barriers**

| Barriers | Papers describing findings (see appendices for details) | Delay Stage | IOM Domain | WHO Health System Building Blocks – Domain |
| --- | --- | --- | --- | --- |
| Self-treatment | Zaidi et al. | 1 | Timeliness |  |
| Non-medical facility visits | Zaidi et al. | 1 | Timeliness |  |
| Non-trauma centers | Zaidi et al.  Rahman et al. | 1 | Timeliness | Health service delivery |
| Inability to seek timely telephone consultation or information (especially in poisoning cases) | Khan et al. | 1 | Timeliness | Health service delivery |
| Longest travel time or driving distance to healthcare facilities | Zaidi et al.  Bhatti et al.  Ihsan et al.  Rahman et al. | 2 | Timeliness | Health service delivery |
| Fragile pre-hospital services with limited geographical coverage – response time delays | Bhatti et al.  Salman et al.  Rahman et al. | 2 | Timeliness | Health service delivery |
| Poor road network | Rahman et al. | 2 | Timeliness |  |
| Traffic congestion due to urbanization | Rahman et al. | 2 | Timeliness |  |
| Inter-hospital transfer delay | Mehmood et al. | 2,3 | Timeliness | Health service delivery |
| Lack of trained pre-hospital staff | Bhatti et al.  Mawani et al.  Rahman et al.  Minhas et al. | 2 | Effectiveness | Health care workforce |
| Lack of coordinated pre-hospital care system | Bhatti et al. | 2 | Safety | Health service delivery |
| Poor communication between first responders and hospitals (No triage system) | Bhatti et al. | 2 | Safety | Health service delivery |
| Districts with lower rates of RTIs have more ambulances. | Ihsan et al. | 2 | Equitable | Health service delivery |
| Inadequate or no provision of first aid or lifesaving interventions during transportation - with no breathing care, bleeding care, intravenous fluids, splints/slab, or cervical collar application or unable to provide patient stabilization at the scene | Bhatti et al.  Mawani et al.  Rahman et al.  Minhas et al. | 2 | Effectiveness | Health service delivery |
| Lack of pre-hospital care management according to standard protocols (especially for traumatic brain injury & traumatic out-of-hospital cardiac arrest) | Bhatti et al.  Rahman et al.  Mawani et al. | 2,3 | Effectiveness | Healthcare workforce |
| Limited or unavailable medical supplies within the ambulances | Bhatti et al.  Mawani et al.  Minhas et al. | 2 | Safety | Essential resources |
| Lack of oxygen supplies in the ambulance | Bhatti et al. | 2 | Safety | Essential resources |
| Unable to provide frequent ambulance maintenance (due to poor road conditions and difficult weather) | Sriram et al. | 2 | Safety | Essential resources |
| Basic healthcare facilities lack the readiness to respond to traumatic emergencies. | Ihsan et al. | 3 | Effectiveness | Health service delivery |
| Lack of defined protocol for the selection of hospitals to transfer trauma patients | Ashraf et al. | 2,3 | Timeliness | Health service delivery |
| The skills, expertise, and equipment infrastructure fell short of recommended standards in several areas in tertiary and secondary care hospitals (especially in pain control and medicine, diagnosis monitoring and safety, neuro-and ENT trauma and rehabilitation) | Khalil et al. | 3 | Effectiveness | Health service delivery |
| Unequal geographical distribution of trauma centers (especially those areas with high rates of RTI) | Ihsan et al. | 3 | Equitable | Health service delivery |
| Unsafe patient referrals and disposition | Salman et al. | 3 | Safety | Health service delivery |
| Delay of antibiotic administration in injured patient | Tahir et al. | 3 | Timeliness | Health service delivery |
| Lack of consultation with experienced physicians in the emergency room (especially for traumatic brain injury patients) | Bhatti et al.  Salman et al.  Rahman et al. | 3 | Effectiveness | Health care workforce |
| Lack of specialized trauma teams in the emergency room | Ihsan et al.  Salman et al.  Hashmi et al. | 3 | Effectiveness | Health care workforce |
| Lack of sub-specialty support like neurosurgeons, cardiothoracic surgery, orthopaedics, toxicologists, interventional radiology & rehabilitation care | Khan et al.  Ihsan et al.  Salman et al.  Arslan et al. | 3 | Effectiveness | Health care workforce |
| Geographical mapping of neurosurgeons and neurosurgery facilities is skewed towards urban centers. | Bakhshi et al. | 3 | Equitable | Health care workforce |
| Initial visits to non-trauma hospitals, where physicians unable to provide ATLS-compliant care | Rahman et al. | 1,3 | Effectiveness | Health care workforce |
| No established trauma care infrastructures or related trauma care networks – like level 1 trauma centers | Ihsan et al.  Salman et al.  Ashraf et al. | 3 | Effectiveness | Health service delivery |
| Lack of ancillary support or allied health staff during the night shift | Saqib et al. | 3 | Safety | Health care workforce |
| Single evening functional operating room | Saqib et al. | 3 | Timeliness | Health service delivery |
| Lack of coordinated in-patient care based on guidelines | Salman et al. | 3 | Effectiveness | Health service delivery |
| Emergency department through-put delays due to incomplete sub-specialty documentation | Salman et al. | 3 | Timeliness | Heath service delivery |
| Insufficient order of point-of-care testing | Salman et al. | 3 | Effectiveness | Health service delivery |
| Lack of 24/7 blood bank, laboratory, and radiology services | Ihsan et al. | 3 | Timeliness | Health service delivery |
| Inadequate stocks of antidotes and resuscitation drugs | Khan et al.  Ihsan et al.  Arslan et al | 3 | Safety | Essential resources |
| Lack of CT scan in the emergency room | Bhatti et al.  Ihsan et al.  Rehman et al. | 3 | Effectiveness | Essential resources |
| Non-functioning operating rooms | Ihsan et al. | 3 | Timeliness | Essential resources |
| No ICU or mechanical ventilator facilities | Ihsan et al. | 3 | Safety | Essential resources |
| Absence of vaccines (e.g., cell-culture vaccines for dog bite cases) | Zaidi et al. | 3 | Effectiveness | Essential resources |
| Unavailability of spinal rehabilitation care beds and physical space | New et al. | 4 | Effectiveness | Essential resources |
| Spinal rehabilitation units have problems with timely access for acute hospital patients. | New et al. | 4 | Timeliness | Health service delivery |
| Low socioeconomic status leads to the inability to get timely, expensive implants to manage open fractures. | Tahir et al. | 4 | Equitable | Essential resources |
| Lack of provision of robust data collection and monitoring | Zaidi et al. | 2, 3, or & 4 | Effectiveness | Health information system |
| Lack of trauma registries | Mehmood et al.  Minhas et al. | 2, 3 or & 4 | Effectiveness | Health information system |
| Limited data storage capacity | Zaidi et al. | 2, 3,4 | Safety | Health information system |
| Provider-based data collection tools/software | Mehmood et al. | 2, 3,4 | Effectiveness | Health information system |
| Poor internet connectivity in remote areas | Zaidi et al. | 2, 3,4 | Timeliness | Health information system |
| Lack of electronic medical records system | Mehmood et al. | 2, 3,4 | Effectiveness | Health information system |
| No formal updated records of poisoning cases and databases of local chemical | Khan et al. | 3 | Safety | Health information system |
| Lack of IT human resources funding - operational costs required travel costs for management and program staff costs for on-site training for m-health applications | Zaidi et al. | 2, 3,4 | Equitable | Health information system |
| Lack of constant IT support | Mehmood et al. | 2, 3,4 | Timeliness | Health Information system |
| Private hospital relies on health insurance or out-of-pocket payments to provide trauma care. | Bhatti et al. | 2,3,4 | Equitable | Heath care Financing |
| Out-of-pocket treatment costs were significantly higher than average for those undergoing CT scans or those utilizing ambulance services. | Bhatti et al. | 2,3,4 | Equitable | Health care Financing |
| Stakeholders reported a need for more regulation and standardization of EMS organizations at the national and regional levels. | Sriram et al. | 2 |  | Healthcare leadership & Governance |
| Social accountability was less established than top-down accountability. | Sriram et al. | 2 |  | Healthcare leadership & Governance |
| Institutional buy-in to adopt trauma registries | Mehmood et al. | 2,3,4 |  | Healthcare Leadership & Governance |
