# Supplementary material for: Access to quality trauma care after injury in Pakistan: a systematic review and narrative synthesis
Source: BMJ Open. 2025 Dec 7;15(12):e101071. doi: 10.1136/bmjopen-2025-101071 (PMC12699741; doi:10.1136/bmjopen-2025-101071)
Supplement: online supplemental file 7 [file bmjopen-15-12-s007.docx]

**Annexure F:**

**Issues found affecting access to Quality Trauma Care After Injury In Pakistan described as Barriers & Facilitators and categorized using the four Delay, IoM, and WHO building blocks**

**Systematic Review & Narrative Synthesis Results – Facilitator**

| Facilitators | Papers describing findings (see appendices for details) | Delay Stage | IOM Domain | WHO Health System Building Blocks – Domain |
| --- | --- | --- | --- | --- |
| Educated and affluent peri-urban and rural population | Zaidi et al. | 1 |  |  |
| Streaming trauma activation protocols | Hashmi et al. | 3 | Timeliness | Health service delivery |
| 24/7 availability of resuscitation, operating rooms, and radiology facilities, interventional radiology, damage control surgeries, and advanced surgical equipment. | Hashmi et al. | 3 | Timeliness | Health service delivery |
| Dedicated trauma care nurse for post-operative care | Hashmi et al. | 3 | Safety | Health care workforce |
| International accreditation of pre-hospital care systems like ISO, JCIA | Sriram et al. | 2 | Safety | Health service delivery |
| Establishing public-private partnerships to focus on providing essential resources and training | Sriram et al. | 2 | Effectiveness | Health leadership & governance |
| Re-enforcing public services in pre-hospital care (In the case of Rescue 1122, 'The Punjab Emergency Services Act' of 2006 mandates the establishment and funding of prehospital emergency services in Punjab Province and sets the rules and regulations by which the service must run) | Sriram et al. | 2 | Equitable | Health leadership & governance |
| On-job training of staff on curricula developed by international institutes | Sriram et al. | 2 | Effectiveness | Health care workforce |
| Recruiting EMTs from nursing with paramedic degrees | Sriram et al. | 2 | Effectiveness | Health care workforce |
| Employing doctors as administrative staff for training and supervisory functions | Sriram et al. | 2 | Effectiveness | Health care workforce |
| Organizational improvement by introducing multidisciplinary integrated trauma care & teams (establishment of 24/7 trauma teams) | Hashmi et al. | 3 | Effectiveness | Health leadership and governance |
| ATLS trained staff | Hashmi et al. | 3 | Effectiveness | Health care workforce |
| Clinical trauma fellowship | Hashmi et al. | 3 | Effectiveness | Health care workforce |
| Resident-centered trauma education | Hashmi et al. | 3 | Effectiveness | Health care workforce |
| Experiential learning – participating in mass casualty incidents | Hashmi et al. | 3 | Effectiveness | Health care workforce |
| Properly functioning emergency surgical interventions | Rizwan et al. | 3 | Safety | Essential resources |
| A constant 24/7 blood bank, radiological and laboratory services | Rizwan et al. | 3 | Safety | Health service delivery |
| Optimizing resource allocations by need assessment and applying quality improvement methods | Rizwan et al. | 3 | Safety | Essential resources |
| Trauma database | Mehmood et al.  Minhas et al.  Hashmi et al. | 2,3,4 | Effectiveness | Health information technologies |
| Low-cost m health-based technologies | Zaidi et al. | 2,3,4 | Equitable | Health information technologies |
