# Supplementary material for: Access to quality trauma care after injury in Pakistan: a systematic review and narrative synthesis
Source: BMJ Open. 2025 Dec 7;15(12):e101071. doi: 10.1136/bmjopen-2025-101071 (PMC12699741; doi:10.1136/bmjopen-2025-101071)
Supplement: online supplemental file 8 [file bmjopen-15-12-s008.docx]

**Annexure G: Studies Reported Initiatives/Interventions to improve access to quality trauma healthcare after injury in Pakistan**

| Studies Reported Initiatives/Interventions to improve access to quality trauma healthcare after injury in Pakistan | |
| --- | --- |
| Papers describing findings | **Initiative/Interventions** |
| Mehmood et al.  2013 | Electronic Karachi Trauma Registry |
| Hashmi et al.  2013 | Trauma quality improvement initiatives |
| Zaidi et al.  2013 | Mobile-based low-cost surveillance system |
| Sriram et al.  2016 | EMS Interventions |
| Minhas et al.  2017 | Trauma registry |
| Rizwan et al.  2018 | Emergency surgical & non-surgical therapeutic interventions |
| Mawani et al.  2018 | Pre-hospital life support interventions |
| Ashraf et al.  2022 | Geospatial modelling |
