# Supplementary material for: Access to quality trauma care after injury in Pakistan: a systematic review and narrative synthesis
Source: BMJ Open. 2025 Dec 7;15(12):e101071. doi: 10.1136/bmjopen-2025-101071 (PMC12699741; doi:10.1136/bmjopen-2025-101071)
Supplement: Supplementary file 9 [file bmjopen-2025-101071.reviewer_comments.pdf]

PEER REVIEW HISTORY

BMJ Open publishes all reviews undertaken for accepted manuscripts. Reviewers are asked to complete a checklist review form and are provided with free text boxes to elaborate on their assessment. These free text comments are reproduced below.

ARTICLE DETAILS

Title (Provisional)

Access to quality trauma care after injury in Pakistan - A systematic review and narrative synthesis

Authors

Atiq, Huba; Rahim , Komal Abdul; Shiekh , Sijal Akhtar; Afzal, Badar; Wajidali , Zabin; Chand, Zaheer Babar; Latif, Asad; Ignatowicz, Agnieszka; Ghalichi, Leila; Chu, Kathryn; Razzak, Junaid A.; Davies, Justine

---

VERSION 1 - REVIEW

**Reviewer**	**1**
**Name**	**Dewi, Ayu Putu Madri**
**Affiliation**	**Amsterdam UMC Location AMC, Epidemiology and Data Science**
**Date**	**06-Mar-2025**
**COI**	**None**

Dear authors,

I am an Open Science researcher, who has checked your manuscript for its adherence to a number of Open Science practices.

Below you find the checklist that I used to assess your manuscript for adherence to a number of these practices.

Based on this assessment, I would suggest the following refinements/additions:

1. *Availability of a protocol: OK*
2. *Pre-registration: OK*
3. *Open Materials: OK*
4. *Open Data: OK*
5. *Open Code: N/A*
6. *Citations: N/A*
7. *Software and tools used are described: OK*
8. *Open access publication: OK*

9. *Reporting checklist provided: OK*
10. *Preprint: Please state explicitly whether and where the preprint of this study can be found.*

**Reviewer**	**2**
**Name**	**van Ditshuizen, Jan**
**Affiliation**	**Erasmus MC**
**Date**	**22-Apr-2025**
**COI**	**None**

Access to quality trauma care after injury in Pakistan - A systematic review and narrative synthesis

General

Nice overview and written down clearly with a passion for organising trauma care in Pakistan. To me 20 studies with 58 barriers, 20 facilitators, and 8 studies providing initiatives/interventions is a lot, especially for a country that is starting to organise trauma care. Where to begin? The authors have managed suyccesfully to create a thorough overview. The one thing I am missing is, how does trauma care relate to other medical acute care disciplines? In the discussion an elaboration on reforming public health care, acute health care or trauma care. How will the trauma patient eventually benefit the most, without huge costs. I wish the authors success with further revising and publication of the paper, and most of all quality trauma care in Pakistan.

Abstract

Methods: IOM is not written out fully, first time abbreviation.
Results: Sentences about the studies that found facilitators and barrierscan be written down more concrete. Suggestion for the last sentence. *According to the WHO Healthcare Building Blocks, most studies (n=15) described "Healthcare service delivery" challenges, identifying 23 barriers and 4 facilitators. Or According to the WHO Healthcare Building Blocks, most studies (n=15) described "Healthcare service delivery" challenges, with 15 studies identifying 23 barriers and 3 studies identifying 4 facilitators.*

Introduction

Second sentence: I understand the sentence, however prevention is another health domain than trauma care. Therefore, a synonym would be more appropriate. Avoided, or a decrease of 2 million death through the provision of ..., result in a survival benefit for 2 million people, less years of life lost through the provision of...

4th paragraph 2nd sentence. An extra reference could be added:
https://www.injuryjournal.com/article/S0020-1383(22)00302-3/fulltext (by Zsolt J. Balogh)

Sentence about Institute of Medicine's (IOM), please add reference.

Last sentence objective: It is unclear to me what preexisting interventions are.

Methods

Add reference about the PICOST framework.

Identification of Studies: With researchers possibly authors is meant? What is iteration of 20%?

Results

4th sentence starts with a number.

Study Design Characteristics: Try to use same usage of n and % throughout the section (n=x, x.x%). 6th paragraph: *with the identification of 37 barriers (n=16 studies) [1, 17-25, 27, 29, 30, 32, 34, 35] and 13 facilitators (n=5 studies)* replace with *with the identification of 37 barriers spread over 16 studies and a total of 13 facilitators reported in 5 studies combined [1, 17-25, 27, 29, 30, 32, 34, 35].*

Usage of word only is subjective.

Discussion

I understand that the focus oif this paper is organising trauma care. It seems that themes at a higher pitch are at play here; economically, educationally, and digitally. Throughout health care. Could you also elaborate on acute health care in general in India? It would provide insight to read something about emergency care in general. Do heart- and neurocare als have these challenges on a broader scale?

**** **The reviewer provided a marked copy with additional comments. Please contact the publisher for full details.** ****

**Reviewer**	**3**
**Name**	**Zamani, Reza**
**Affiliation**	**University of Exeter**
**Date**	**26-Aug-2025**
**COI**	**None**

This is well prepared and constructed systematic review in this crucial area of trauma care. The article identifies a number of issues of traumatic injury care, some of which are shared with other LMICs. To improve, it would be useful to address the following issues.

the title has the word 'quality'. This seems to be redundant, since, as the article shows, there are significant challenges in Pakistan to provide high quality care.

other issues to consider is shown below indicate by page (as shown by PDF, not article page) and line number.

• P7 8; causes

• P6 53; however, at the start of a paragraph! not recommended.

• P8 9; 60% should be qualified against total population of LMICs. It is only meaningful if LMICs and other richer countries have each 50% of the world population

• P8 35; mapped or map?

• P9 9; why until 2023 and not 2025?

• P9 18-24; difficult sentence structure

• P9; In search databases, not sure why thesis and grey website are used for this review. It can only lower the quality of the article as these resources are not peer reviewed. However, authoritative sources such as WHO could surely be used.

• P9; The syntax in search databases should be reviewed

• P10 8-10; why? No justification given

• P11; Under study population section, it would be helpful to provide cumulative stats on population number, age range and mean, and other demographic distributions such as education attainment, socioeconomic status, rural vs urban residency etc.

• P12 45; full stop missing.

• P15 57; should it be emergency ward or room?

• P16 39; consistent with

• P16 39-43; you have not really discussed any of these issues above in any details, so not convinced if you could claim your findings are consistent with…. Perhaps discuss the notions of fear, distrust of the healthcare system, lack of awareness, underestimation of the severity of injuries, financial constraints, religious beliefs, and seeking care from traditional healers in more details based on the selected literature to justify your statement.

• P17 3; here and elsewhere, state Pakistan, not 'our country'

• P17 48: under limitation of study, no mention of CASP results and its analysis is stated.

• P17 56; IOM or IoM?

• P27 1; figure 2 is not referred to in the text; not convinced that this composite figure is adding anything to the paper either.

VERSION 1 - AUTHOR RESPONSE

B. Reviewer: 1 - Ms. Ayu Putu Madri Dewi, Amsterdam UMC, Location AMC

1. *I have some feedback related to open science practices. Please find attached the feedback details.*

   Dear authors,
   I am an Open Science researcher, who has checked your manuscript for its adherence to a number of Open Science practices.
   Below you find the checklist that I used to assess your manuscript for adherence to a number of
   these practices.
   Based on this assessment, I would suggest the following refinements/additions:
   1. Availability of a protocol: OK
   2. Pre-registration: OK
   3. Open Materials: OK
   4. Open Data: OK
   5. Open Code: N/A
   6. Citations: N/A
   7. Software and tools used are described: OK
   8. Open access publication: OK
   9. Reporting checklist provided: OK
   10. Preprint: Please state explicitly whether and where the preprint of this study can be found.

   Answer: We confirm that no preprint of this study has been posted. We have now made this explicit in the manuscript by adding a statement to the Data Availability section (page 9).

C. Reviewer: 2 - Dr. Jan van Ditshuizen, Erasmus MC

1. *General: Nice overview and written down clearly with a passion for organising trauma care in Pakistan. To me 20 studies with 58 barriers, 20 facilitators, and 8 studies providing initiatives/interventions is a lot, especially for a country that is starting to organise trauma care. Where to begin? The authors have managed successfully to create a thorough overview.*

   The one thing I am missing is how trauma care relate to other medical acute care disciplines? In the discussion, an elaboration on reforming public health care, acute health care, or trauma care. How will the trauma patient eventually benefit the most, without huge costs? I wish the authors success with further revising and publication of the paper, and most of all, quality trauma care in Pakistan.

   I understand that the focus of this paper is on organising trauma care. It seems that themes at a higher pitch are at play here: economically, educationally, and digitally. Throughout health care. Could you also elaborate on acute health care in general in India? It would provide insight to read something about emergency care in general. Do Heart and Neurocare also have these challenges on a broader scale?

   Answer: In the revised Discussion, we have now elaborated on how the barriers identified in trauma care overlap with other time-sensitive illnesses in Pakistan. Specifically, we highlight that many challenges, such as limited prehospital services, shortage of specialized workforce, and lack

of essential resources, are not unique to trauma but also affect patients with cardiac arrest, stroke, neonatal and obstetric emergencies, and sepsis. We have emphasized that strengthening trauma care should not occur in isolation, but rather as part of broader acute healthcare reform. To reflect this, we have added the following text to the Discussion (page 17):

"Moreover, many of the identified barriers, such as limited prehospital services, lack of trained specialized workforce, lack of essential resources, and registries, are not unique to providing quality trauma care but also affect patients with other time-sensitive illnesses like cardiac arrest, stroke, neonatal and obstetric emergencies, and sepsis [64-69]. Therefore, strengthening the quality of trauma care within Pakistan's health system should not be done in isolation; rather, the need offers an opportunity to reform the quality of the healthcare system broadly, especially for acute care [10]. Similar challenges to what we have found have been documented in nearby India, where inadequate prehospital care and communication, frequent medical errors, delays in transfusion and imaging, and limited surgical capacity hinder trauma resuscitation [70-72]. Therefore, this health systems-oriented approach is consistent with global calls to integrate quality trauma care into broader emergency and acute care reform in low- and middle-income countries [12]".

2. *Abstract*
    a. *Methods: IOM is not written out fully, first time abbreviation.*

       Answer: Corrected to Institute of Medicine (IOM) (page 4).

    b. *Results: Sentences about the studies that found facilitators and barriers can be written down more concrete. Suggestion for the last sentence. According to the WHO Healthcare Building Blocks, most studies (n=15) described "Healthcare service delivery" challenges, identifying 23 barriers and 4 facilitators. Or According to the WHO Healthcare Building Blocks, most studies (n=15) described "Healthcare service delivery" challenges, with 15 studies identifying 23 barriers and 3 studies identifying 4 facilitators.*
       Answer: In the revised Results section, we have made the reporting of barriers and facilitators more concrete by providing both the number of studies and the number of barriers/facilitators identified. For example, under the WHO Healthcare Building Blocks, we now state: "According to the WHO health system building blocks, most studies (n=15) described challenges in healthcare service delivery, with these 15 studies identifying 23 barriers and 3 studies identifying 4 facilitators." This adjustment has been applied consistently across the Results to improve clarity and precision (page 4 and 5).

3. Introduction
    a. *Second sentence: I understand the sentence, however prevention is another health domain than trauma care. Therefore, a synonym would be more appropriate. Avoided, or a decrease of 2 million death through the provision of ..., result in a survival benefit for 2 million people, less years of life lost through the provision of...*

       Answer: We agree that the term "prevented" may imply injury prevention rather than trauma care, and therefore, we have revised the sentence as follows:

“The burden of injury is disproportionately higher in low- and middle-income countries (LMICs) than in high-income countries, and it is estimated that 2 million deaths per year could be averted through the provision of adequate trauma care in LMICs.” (page 6)

b. *4th paragraph 2nd sentence. An extra reference could be added: https://www.injuryjournal.com/article/S0020-1383(22)00302-3/fulltext (by Zsolt J. Balogh)*

   Answer: We added the reference (page 6).

c. *Sentence about Institute of Medicine's (IOM), please add reference.*

   Answer: We added the reference (page 7).

d. *Last sentence objective: It is unclear to me what preexisting interventions are.*

   Answer: By “pre-existing interventions,” we intended to refer to initiatives and interventions that have already been reported in the literature from Pakistan (page 7). For example, trauma registry, trauma quality improvement initiatives, and pre-hospital life support interventions. We submitted a detail list of pre-existing initiatives or interventions as Supplementary File 7 Annexure H.

4. Methods
   a. *Add a reference to the PICOST framework*.
      Answer: Reference added as follows (page 8)

      - Schnaubelt, S., et al., International facets of the ‘chain of survival’ for out-of-hospital and in-hospital cardiac arrest – A scoping review. Resuscitation Plus, 2024. 19: p. 100689.

   b. *Identification of Studies: With researchers possibly authors, is meant? What is the iteration of 20%?*

      Answer: By “researchers,” we were referring to the authors of this review (HA and KAR). To improve clarity, we have revised the sentence to specify that both authors independently reviewed and compared their results for a random 20% of the articles to ensure consistency in applying the inclusion and exclusion criteria (page 8).

5. Results
   a. *4th sentence starts with a number.*

      Answer: Corrected to Seventeen from 17.

   b. *Design Characteristics: Try to use the same usage of n and % throughout the section (n=x, x.x%).*

      Answer: Corrected

c. *6th paragraph: with the identification of 37 barriers (n=16 studies) [1, 17-25, 27, 29, 30, 32, 34, 35] and 13 facilitators (n=5 studies) replace with with the identification of 37 barriers spread over 16 studies and a total of 13 facilitators reported in 5 studies combined [1, 17-25, 27, 29, 30, 32, 34, 35]. Usage of word only is subjective.*

   Answer: Corrected

E. Reviewer: 3 Dr. Reza Zamani, University of Exeter

1. *This is a well-prepared and constructed systematic review in this crucial area of trauma care. The article identifies a number of issues of traumatic injury care, some of which are shared with other LMICs. To improve, it would be useful to address the following issues. The title has the word 'quality'. This seems to be redundant, since, as the article shows, there are significant challenges in Pakistan to provide high-quality care.*
   Answer: We agree that there are major challenges in Pakistan in delivering high-quality trauma care. The word "quality" in the title emphasizes that this review not only examines access to trauma care services but also assesses the standards of care delivered holistically. We applied the Four Delays framework to assess access. At each delay, we examined barriers and facilitators to providing quality trauma care, guided by the Institute of Medicine quality domains and the WHO health system building blocks.

   Our findings are also consistent with the Lancet Global Health Commission on High-Quality Health Systems, which reported that "nearly 8 million lives are lost annually in LMICs due to treatable conditions, 60% of these deaths occur among people who accessed care but received poor-quality services, highlighting that poor quality is now a bigger barrier to reducing mortality than insufficient access".

   We believe it is important that the title reflects both aspects of access and quality. We have therefore retained the word "quality" to highlight this distinction.

2. *P7 8; causes*

   Answer: Corrected

3. *P6 53; however, at the start of a paragraph! Not recommended.*

   Answer: Corrected

4. *P8 9; 60% should be qualified against the total population of LMICs. It is only meaningful if LMICs and other richer countries each have 50% of the world's population.*

   Answer:
   We apologies for the lack of clarity and have adjusted this text to highlight better the burden of lack of access to quality care in LMICs, as below: These challenges reflect the fragile and non-integrated healthcare system in the country and are consistent with the findings of the Lancet Global Health Commission on High-Quality Health Systems. The Commission estimated that each year, 8.6 million people in LMICs die from conditions that should be treatable within health systems. Approximately 3.6 million of these deaths are due to lack of access, while 5.0

million (nearly 60%) occur among those who accessed care but received poor-quality services (12).

- *Kruk, M.E., et al., High-quality health systems in the Sustainable Development Goals era: time for a revolution. The Lancet Global Health, 2018. 6(11): p. e1196-e1252.*

5. *P8 35; mapped or map?*

   Answer: Corrected to mapped

6. *P9 9; why until 2023 and not 2025?*
   Answer: The literature search commenced in February 2024; we searched 10 years prior to this date to ensure we were capturing studies that were likely to be still informing practice and policy in Pakistan. We submitted the paper for peer review in February 2024 to BMJ Open, 12 months after the search commenced. The peer review process itself has taken 8 months, which explains the apparent time lag.

7. *P9 18-24; difficult sentence structure*

   Answer:

   This has been revised to: Grey literature was searched through the first 20 pages of Google Scholar and by targeting local websites, such as the Higher Education Commission, the Pakistan Medical Association, and local thesis repositories relevant to trauma care in Pakistan. We also reviewed non-published reports, national and international databases (e.g., WHO), guidelines, and factsheets identified as relevant by the authors (page 8).

8. *P9; In search databases, not sure why thesis and grey website are used for this review. It can only lower the quality of the article, as these resources are not peer-reviewed. However, authoritative sources such as the WHO could surely be used.*

   Answer: We recognize that grey literature, including theses and non-peer-reviewed reports, may have limitations in terms of quality. However, given our consideration that many interventions are not written up as formal scientific publications, especially in Pakistan, we felt it was important to conduct a comprehensive review that also considered grey literature. Including these sources allowed us to capture potentially relevant information, such as unpublished reviews, national reports, or factual data not available in peer-reviewed journals. Fifteen percent of the included studies were drawn from grey literature, identified through screening the first 20 pages of Google search results. Based on the CASP checklist, the methodological quality of these studies was approximately comparable to that of the peer-reviewed studies. We have explained this rationale in our Methods and Results (pages 8 and 10) and included a quality assessment in the Supplementary File 2, Annexure B: Studies Quality Assessment Checklist.

9. *P9; The syntax in search databases should be reviewed*

   Answer: We reviewed the syntax again and provided all database search strategies as a Supplementary File 1, Annexure A: Search Database and Search Strategy.

10. *P10 8-10; why? No justification given*

    Answer: We aimed to conduct a comprehensive mapping of available evidence and generate insights rather than to perform a meta-analysis of effect sizes. Quality appraisal was performed using the CASP checklists to transparently highlight methodological strengths and limitations, but all studies were retained to ensure that potentially valuable contextual information was not lost. This approach is consistent with the narrative synthesis methodology, which emphasizes inclusivity of diverse sources while accounting for quality in the interpretation of findings. We added the justification in main manuscript (page 9).

11. *P11; Under the study population section, it would be helpful to provide cumulative statistics on population number, age range, and mean, as well as other demographic distributions such as education attainment, socioeconomic status, rural vs urban residency, etc.*

    Answer: In the revised manuscript, we have added a cumulative summary of the study populations across all included studies. A total of 11 patient-related studies were included in this systematic review, comprising 15,060 participants, with individual study sample sizes ranging from 32 to 6,212. The mean age across nine studies was 34.5 years, with participants ranging from 1 to 89 years of age. Gender distribution was reported in all included studies (n=11), with 11,628 males and 3,294 females. Information on education level and socioeconomic status was not reported in any of the studies.

    These details are presented in the Table and summarized in the Study Populations section (page 10 and supplementary file 4 Annexure D).

    Table: Summary of Study Population Across Included Studies

**Variables (n= number of studies reported)**	**Cumulative Total (n)**	**Mean/Range/ Percentage**
Total participants (n=11)	15,060	32-6,212
Age (years) (n=9)	14,922	34.5 years (1-89 years)
Gender (n=11)	14,922	Male – 11,628 (77.9%)<br>Female – 3,294 (22.07%)
Education	NA	Not reported in any study
Socioeconomic status	NA	Not reported in any study

12. *P12 45; full stop missing.*

    Answer: Formatted

13. *P15 57; should it be the emergency ward or the room?*

    Answer: Corrected to emergency room.

14. P16 39; consistent with

Answer: Corrected

15. *P16 39-43; you have not really discussed any of these issues above in any detail, so not convinced if you could claim your findings are consistent with…. Perhaps discuss the notions of fear, distrust of the healthcare system, lack of awareness, underestimation of the severity of injuries, financial constraints, religious beliefs, and seeking care from traditional healers in more detail based on the selected literature to justify your statement.*

    Answer: Thank you for highlighting this. In our systematic review, we identified key barriers at Delay Stage 1, including self-treatment, non-medical facility visits, and an inability to seek timely telephonic care. We acknowledge that the broader literature from other LMICs also reports additional factors, such as fear and distrust of the healthcare system, financial constraints, religious beliefs, and underestimation of injury severity, that contribute to delays in seeking formal care. We have now expanded the discussion to reflect these factors and referenced relevant studies accordingly (page 16).

16. *P17 3; here and elsewhere, state Pakistan, not 'our country'*

    Answer: Corrected

17. *P17 48: Under the limitation of the study, no mention of CASP results and their analysis is stated.*

    Answer: We have described the CASP quality appraisal results in the Risk of Bias section. In response to this comment, we have also added a sentence under the Limitations section to acknowledge that the included studies were of variable quality according to the CASP assessment, which may have influenced the strength of our conclusions (page 17).

18. *P17 56; IOM or IoM?*

    Answer: Corrected to IOM

19. *P27 1; Figure 2 is not referred to in the text; I am not convinced that this composite figure adds anything to the paper either.*

    Answer: Figure 2 is intended to provide a visual, at-a-glance representation of the distribution of available literature across the Four Delays Framework, the Institute of Medicine quality domains, and the WHO health system building blocks. We believe that this figure adds value by helping readers quickly understand where the evidence is concentrated and where major gaps exist, complementing the detailed results in the text.

    To address the reviewer's concern, we have now explicitly referred to Figure 2 in the Results section (pages 11) and clarified its role as a summary visualization of the evidence mapping.
